# The prognostic value of frailty criteria in older adults: a systematic review and meta-analysis

**DOI:** 10.1007/s40520-026-03458-5

**Published:** 2026-08-03

**Authors:** Konstantinos Prokopidis, Francesco Saverio Ragusa, Roberta Bonica, Alessandro D’Aleo, Mathias Schlögl, Gustavo Duque, Mario Barbagallo, Nicola Veronese

**Affiliations:** 1 Department of Musculoskeletal Ageing and Science, Institute of Life Course and Medical Sciences, University of Liverpool, Liverpool, UK; 2 Department of Health Promotion, Maternal and Infant Care, Internal Medicine and Medical Specialties, “G. D’Alessandro”-PROMISE-University of Palermo, Palermo, Italy; 3 Saint Camillus International, University of Health Sciences, Rome, Italy; 4 Chief Medical Officer Geriatric Medicine, Clinic Barmelweid AG, Barmelweid, Switzerland; 5 Bone, Muscle & Geroscience Group, Research Institute of the McGill University Health Centre, Montreal, QC Canada; 6 Department of Medicine, Dr. Joseph Kaufmann Chair in Geriatric Medicine, McGill University, Montreal, QC Canada

**Keywords:** Frailty, Mortality, Hospitalization, Cardiovascular disease

## Abstract

**Introduction:**

Frailty, a prevalent geriatric syndrome, increases susceptibility to adverse outcomes like mortality, cardiovascular disease, and hospitalization. This study evaluated the Fried frailty phenotype and its components’ association with clinical outcomes, focusing on mortality.

**Methods:**

We conducted a systematic review and meta-analysis of studies on individuals aged 50 or more from databases searched up to October 2024. Included studies assessed at least one frailty criterion (weakness, slowness, exhaustion, physical inactivity, weight loss) and reported hazard ratios (HRs) for mortality or other outcomes. Random-effects models and subgroup analyses (e.g., heart failure, chronic kidney disease) were employed.

**Results:**

From 3445 articles, 36 studies were included. All frailty components are significantly associated with all-cause mortality: weakness (HR: 1.44, 95%CI: 1.30–1.59, p < 0.01), slowness (HR: 1.75, 95%CI: 1.56–1.95, p < 0.01), exhaustion (HR: 1.47, 95%CI: 1.29–1.68, p < 0.01), physical inactivity (HR: 1.72, 95%CI: 1.55–1.92, p < 0.01), and weight loss (HR: 1.56, 95%CI: 1.31–1.84, p < 0.01). Slowness, weakness, and physical inactivity were linked to cardiovascular mortality (HRs: 1.97, 1.45, 1.26, respectively, p < 0.01), while slowness and exhaustion were also linked to an increased risk of hospitalization (HRs: 1.46, 1.25, p < 0.01). Slowness was associated with a two-fold higher mortality risk (I2 = 57%, HR: 1.99, 95%CI: 1.73–2.30, p < 0.01). Subgroup analyses confirmed these links in heart failure and chronic kidney disease patients.

**Conclusion:**

Frailty components, especially weakness and slowness, are significantly associated with mortality and related outcomes, highlighting the urgency of early frailty detection to improve patient prognosis.

**Graphical abstract:**

**Figure:**
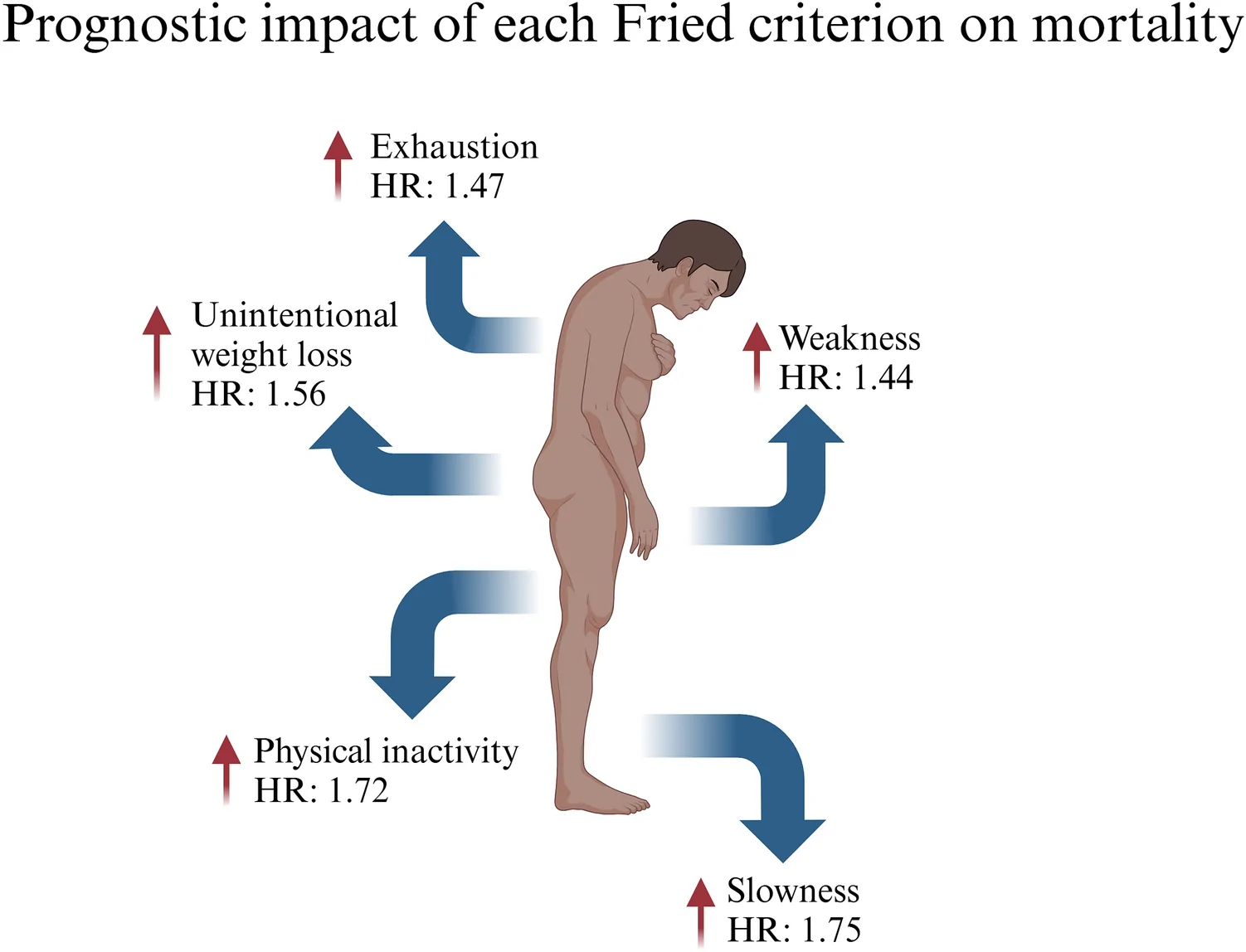

This systematic review and meta-analysis of 36 studies demonstrates that the components of frailty, when considered individually, are significantly associated with an increased mortality risk; weakness (HR: 1.44), slowness (HR: 1.75), exhaustion (HR: 1.47), physical inactivity (HR: 1.72), and unintentional weight loss (HR: 1.56). Slowness and weakness are also associated with cardiovascular mortality and hospitalization, with slowness associated with a two-fold higher mortality risk in community-dwelling older adults (HR: 1.99). These robust associations, confirmed in heart failure and chronic kidney disease subgroups, highlight frailty's critical role in adverse outcomes, urging early identification and intervention to enhance patient care and reduce clinical burden.

**Supplementary Information:**

The online version contains supplementary material available at https://doi.org/10.1007/s40520-026-03458-5.

## Introduction

Frailty is a prevalent and significant geriatric syndrome, defined by age-related reductions in physiological reserve and function across multiple organ systems, resulting in heightened susceptibility to adverse health outcomes [1]. Due to the aging population increment even frailty incidence all over the world is increasing: an Australian systematic review and meta-analysis, incorporating data from over 120,000 older adults across 28 countries, estimated the incidence rates of frailty and prefrailty at 43.4 and 150.6 new cases per 1,000 person-years, respectively [2]. By 2043, the years lived with frailty are projected to rise from 3.7 to 4.0 years for women and from 1.9 to 2.1 years for men, spanning all educational levels [2]. At the same time frailty increased with age, affecting 10% of adults aged 50–64 and 43.7% of adults aged ≥ 65 [3].

One of the most widely used tools for defining frailty are criteria proposed by Fried and colleagues. With this definition, frailty is characterized as a clinical syndrome identified by the presence of three or more of the five widely used criteria, i.e., unintentional weight loss (> 10 pounds over the past year), self-reported fatigue, reduced grip strength—below the lowest quintile for gender and body mass index (BMI) (weakness), decreased walking speed/15 feet -slowest 20% by gender and height- (slowness), and low levels of physical activity -lowest 20% among males and females- [4]. This definition and the frailty index are probably the most used, to identify older people who may be considered frail [5].

Frailty was not only significantly associated with a higher risk of all-cause mortality but also emerged as a strong predictor of cause-specific mortality from cardiovascular disease (CVD), cancer, and respiratory illnesses in older adults [6]. Higher levels of frailty were also linked to an elevated risk of hospitalization for falls, fractures of any kind, and specifically hip fractures [7]. Previous studies confirmed the role of individual frailty phenotype components in mortality prediction: a British study of 502,536 older adults with and without type 2 diabetes mellitus (T2DM) revealed how in both populations, slow walking pace showed the strongest association with all-cause mortality among all frailty components and overall frailty status [8]. In this paper the identification of T2DM was based on self-report physician-diagnosed and the overall prevalence of T2DM they reported was slightly lower than the general population. Another Spanish study of 342 individuals, where among the components of Fried’s criteria, only physical activity and gait speed were independently associated with mortality [9], but in this work frailty phenotype was determined in the convalescence phase of acute coronary syndrome and the sample was not large. Also an American study of 667 kidney transplant recipients revealed how only exhaustion and slowed walking speed emerged as having an association with post- transplant mortality [10], however, since the study was conducted at a single center, the findings should be interpreted with caution and considered within the context of that specific study population.

This is, to our knowledge, one of the first systematic review and meta-analysis quantifying the association of each frailty component with multiple outcomes, including all-cause mortality, cardiovascular mortality, and hospitalization. In addition, our subgroup analyses in high-risk populations such as heart failure and chronic kidney disease extend the importance of this work, providing valuable insight in clinical populations with increased frailty risk. Rather than proposing a new frailty framework, our objective was to refine and strengthen the evidence on the relative prognostic importance of established frailty components using contemporary data and larger pooled samples. The consistency and magnitude of the associations observed—particularly for slowness and weakness—provide clinically relevant insights that support targeted frailty assessment in practice.

## Methods

### Search strategy

Two independent reviewers (KP, FSR) searched PubMed, Scopus, Web of Science and Cochrane Library from inception until October 2024, using the search strategy outlined in Table S1. The study is registered in PROSPERO.

### Inclusion and exclusion criteria

Studies were included based on the following criteria: (i) longitudinal (prospective or retrospective) studies with individuals 50 years old and above irrespective of health status; [11] (ii) at least one frailty criterion was assessed, for which, cut-off points were identical to the one’s displayed based on the criteria proposed by Fried; (iii) an outcome of all-cause mortality, cardiovascular mortality, cardiovascular events, hospitalization, or falls. Studies were excluded if: (i) did not report data that could be meta-analysed; (ii) did not have a full text; (iii) were not written in English.

### Data extraction

Two investigators (KP, FSR) extracted data independently, including the name of first author, year of publication, country of origin, health status, patient characteristics (age, sample size, sex, BMI), study duration, confounders used in adjusted analysis, and outcomes of interest. Data screening and data extraction were performed using Microsoft Excel.

Discrepancies between authors were resolved by a third investigator (NV). This systematic review protocol was registered in the PROSPERO international prospective register of systematic reviews (registration number: CRD42024598977). The registration was completed on November 4th, prior to the commencement of the review, to ensure methodological transparency and reduce the risk of reporting bias.

### Risk of bias assessment

The Newcastle–Ottawa Scale (NOS) assessment was used by two authors (ADL, RB) to evaluate risk of bias of the included studies [12]. The NOS assigns up to a maximum of nine points for the least risk of bias in three domains: 1) selection of study groups (four points); 2) comparability of groups (two points); and 3) ascertainment of exposure and outcomes (three points) for case–control and cohort studies. Studies with 8–9 points were considered of high quality, those with 6–7 points of moderate quality, and those with 5 and below of low quality. Discrepancies between authors were resolved by a third investigator (KP).

### Statistical analysis

Outcomes between older adults meeting at least a frailty criterion vs. its reference were compared using hazard ratios (HR). Quantitative data were treated as continuous. For studies presenting interquartile ranges (IQR), the formula ‘standard deviation (SD) = width of IQR/1.35’ approximated missing SDs [13]. Statistical significance was determined through the random-effects model and inverse-variance method. Evaluation of heterogeneity among outcome measures across studies employed a 95% confidence interval (95%CI) overlap, Cochran’s Q (chi-square test), and I^2^. Data was categorized as having low (30%–49%), moderate (50%–74%), or high (75% and above) heterogeneity. Subgroup analyses were based on sufficient studies reporting community-dwelling adults and those with a specific comorbidity. Publication bias was assessed via Egger’s test and by visually inspecting funnel plots, while the trim-and-fill method was employed to estimate unbiased effect sizes. Random-effects meta-regressions based on age, BMI, and sex were conducted to explore sources of heterogeneity. The synthesis of meta-analyses was conducted through R software using ‘meta’ package (version 4.3.1). A p value of < 0.05 was set as a significant result. All the characteristic of analyses performed were reported in supplementary material (Table S2).

## Results

### Search results

**Fig. 1:**
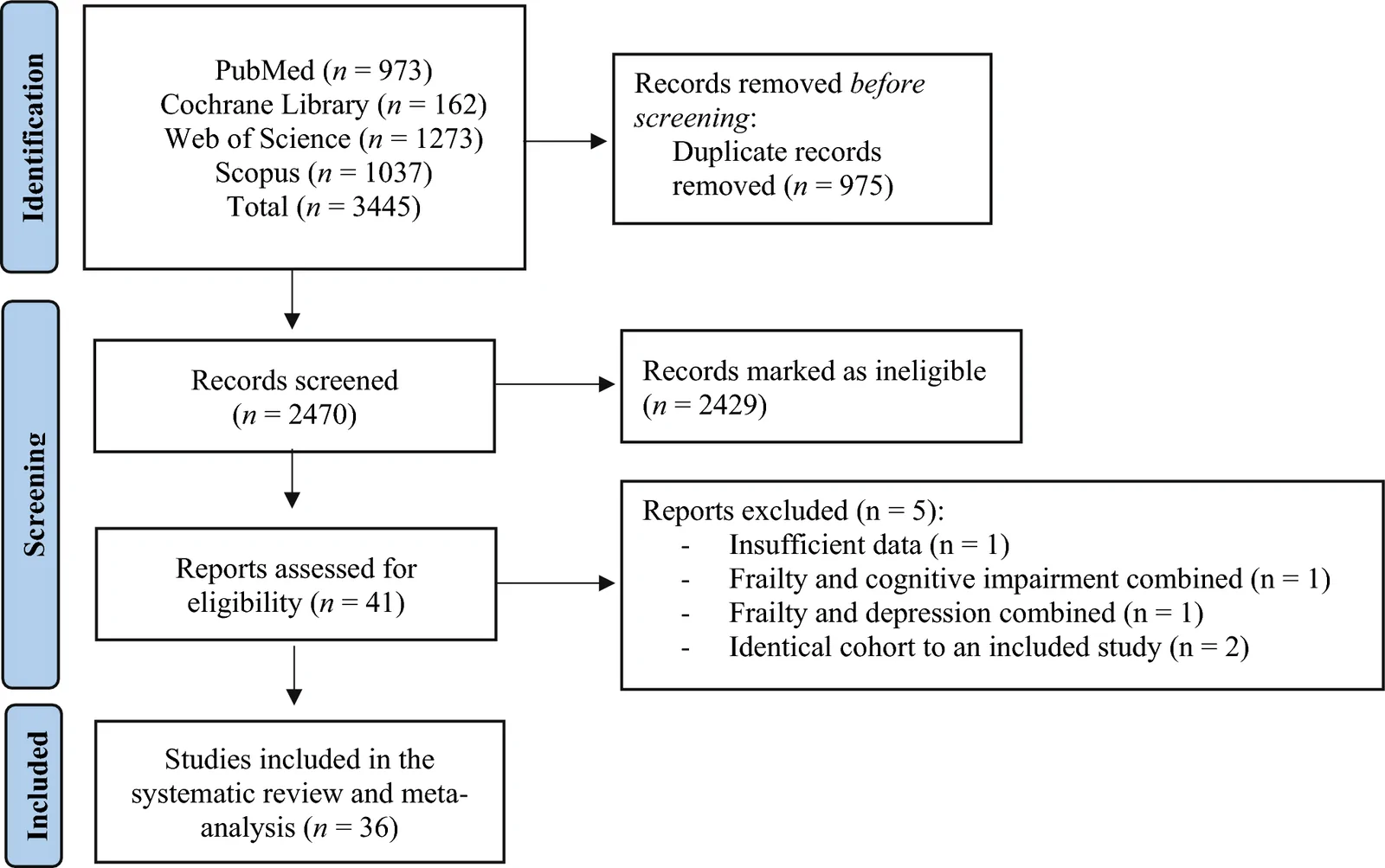
Flowchart of the employed search strategy

Our initial search yielded 3445 total articles. Following the removal of duplicates, 2464 records were screened, for which, 2429 were marked as ineligible according to the inclusion criteria based on their title and abstracts. Thirty-five articles were eventually assessed, where two had identical cohorts with studies that were more recent and eligible to be included [8, 14], one study used frailty combined with cognitive impairment [15], one study used frailty combined with depression [16], while one study had insufficient data [17]. The present systematic review was conducted in accordance with the Preferred Reporting Items for Systematic Reviews and Meta-Analyses (PRISMA) guidelines. The study selection process is summarized in Fig. 1, which reports the PRISMA flow diagram, and the review protocol was prospectively registered in PROSPERO prior to data extraction. In total, 36 studies were included in this systematic review and meta-analysis [8, 15–51]. Older adults’ age ranged between 51 to 86, with the majority of the studies examining community-dwelling, or adults with chronic kidney disease or undergoing hemodialysis, and adults with heart failure. The prevalence of adults diagnosed with frailty ranged from 1.3% to 58.6% across the included studies. Comprehensive characteristics of the included studies are presented in Table 1. In this table studies are listed in chronological order of publication to facilitate comparison across time and highlight the evolution of evidence. For each study, we report key characteristics relevant to the meta-analysis, including participants’ health status, primary outcome assessed, mean age, sex distribution, sample size, proportion of participants with frailty, follow-up duration, country, and adjusted covariates. This structure allows readers to easily compare study designs, populations, and analytical adjustments across the included studies.

**Table 1:** Characteristics of the included studies

Study	Health status	Outcome	Age, mean (SD)	Percentage of women	Sample size (m/f)	% of participants with frailty	Follow-up	Country	Adjusted covariates
[18]	DSM-IV Depression	Mortality	70.7 (7.4)	66%	378 (128/250)	27%	6 years	The Netherlands	Age, sex, level of education, smoking, alcohol use, physical activity, insomnia, depressive symptom severity, cognitive functioning, number of chronic somatic diseases, and number of prescribed medications
[19]	Community-dwelling	Hospitalization	65.8 (6.0)	27.5%	5169 (3750/1419)	2.1%	15.2 months	United Kingdom	Age at baseline and sex
[21]	Chronic kidney disease	Mortality	Median age: 51 (non-frail), 64 (frail)	32%	109 (73/36)	28.4%	3.3 years	Canada	Age, sex, albumin, hemoglobin and comorbidity count
[22]	Community-dwelling	Mortality	75.4 (5.9)	56%	1531 (664/867)	4.6%	5.5 years	Spain	Sarcopenia, age, sex, Charlson Index
[23]	Community-dwelling	Mortality	63.86 (2.8)	55.9%	91,906 (40,518/51,388)	1.63%	12.5 years	UK	Age, sex, Towsend deprivation index, educational levels, country, ethnicity, smoking status, BMI, use of antihypertensive or lipid-lowering medication, insulin, chronic conditions
[24]	Fibrotic interstitial lung disease	Mortality	69 (11)	44.49%	463 (257/206)	26% frail, 56% pre-frail	Median 1.71 years	Canada	Age, sex, lung function
[25]	Chronic kidney disease	Atherosclerotic events, Incident heart failure, Mortality, Cardiovascular mortality, non-cardiovascular mortality	62.0 (10.5)	46%	2539 (1361/1178)	12% frail, 51% prefrail	Median 11.4 years	USA	Age, sex, race, ethnicity, education, marital status, smoking, BMI, systolic BP, diabetes mellitus, cardiovascular disease, use of angiotensin-converting enzyme inhibitor/angiotensin receptor blockers, aspirin, or statins, low-density lipoprotein (LDL) cholesterol, eGFR, and proteinuria
[26]	Heart Failure-LVAD	Length of stay, In-hospital mortality, Mortality	58 (11.9)	25%	75 (56/19)	58.6%	Median 314 days	USA	-
[27]	Community-dwelling or Patient	Mortality	Males: 74 (69.79) Females: 72 (68.78)	46%	4996 (2691/2305)	14%	Median 17.3 months	52 countries	-
[28]	Heart Failure	Mortality, Heart failure hospitalisation	61.2 (14.3)	32.42%	3429 (2283/1146)	21% frail, 61% pre-frail	Median 3.1 years	Canada	Country income level; age; sex; education; HF aetiology; left ventricular ejection fraction; diabetes; tobacco and alcohol use; New York Heart Association functional class; HF medication use; blood pressure; and haemoglobin, sodium, and creatinine c
[30]	Undergoing hemodialysis	Mortality, Emergency Visits, Hospitalisation, CVD events, Falls	69	51.3%	150 (73/77)	34.67%	12 months	China	Age, gender, albumin
[31]	Hypertension	Mortality	70.03 (10.14)	52.53%	2117 (1005/1112)	17.81%	130.1 months	USA	Age, sex, race, education, poverty–income ratio, smoking, alcohol, diabetes, arthritis, congestive heart failure, coronary heart disease, stroke, overweight, cancer or malignancy, chronic obstructive pulmonary disease, chronic kidney disease, and taking medicine for hypertension
[34]	Chronic obstructive pulmonary disease	Mortality, Hospitalization	86	22%	309 (241/68)	49.8%	1 years	China	Age, gender, Charlson comorbidity index, medication, GOLD severity, moderate-to-severe exacerbation history, and COPD Assessment Test
[33]	Community-dwelling	Cardiovascular disease, Cardiovascular mortality	65.8 (7.6)	65.2%	5015 (1747/3268)	3.7% frail, 46.2% pre-frail	10 years	Singapore	Age, sex, race, education, poverty–income ratio, smoking, alcohol, diabetes, arthritis, congestive heart failure, coronary heart disease, stroke, overweight, cancer or malignancy, chronic obstructive pulmonary disease, chronic kidney disease, and taking medicine for hypertension
[35]	Community-dwelling	Mortality	Not explicitly mentioned in summary	48.8%	1724 (836/888)	11.3% frail; 32.5% pre-frail	8 years	China	Age, gender, Charlson comorbidity index, medication, GOLD severity, moderate-to-severe exacerbation history, and COPD Assessment Test
[36]	Heart failure with preserved ejection fraction	Hospitalization	75.5 (6.52)	57.3%	520 (222/298)	27.9% frail	3 years	China	Age, sex, educational levels, marital status, live place, current smoking, alcohol drinking, activities, chronic diseases, BMI
[8]	Type 2 diabetes	Mortality, Cardiovascular mortality	65.3 (7.8)	55%	6000 (2700/3300)	30%	Median: 12.5 years	United Kingdom	Age, sex
[37]	Community-dwelling	Mortality	With frailty: 74.8 (6.4); Without frailty: 72.8 (5.6)	48.42%	1174 (383/791)	24.7%	5 years	Chile	Sex, age, polypharmacy, Athens Insomnia Scale, MMSE, LVEF, BADL, and IADL
[38]	Community-dwelling	Disability, Falls, Mortality	77.95 (4.36)	0%	1198 (1198/0)	14% frail, 54% prefrail	3 years	United Kingdom	Age, sex, ethnicity, smoking, BMI, systolic blood pressure, lipid lowering medication, blood pressure medications, LDL cholesterol, employment, alcohol, smoking deprivation, prevalent CVD, prevalent cancer
[39]	Cognitive impairment	Mortality	79 (7.4)	62%	1154 (437/717)	36.4% frail, 45.9% prefrail	2 years	United Kingdom	Age, sex, MMSE, socio economic status
[40]	Graft-versus-host disease	Non-relapse mortality, Mortality	-	40.6%	399 (237/162)	32% frail	10 years	USA	Age
[41]	Heart failure	Mortality, Rehospitalization	85.2 (7.3)	61%	497 (211/286)	57.5%	1 year	Spain	Age, sex, living situation (care home, residential homes, nursing homes and long-stay hospitals, or own home), cognitive impairment (MMSE score) and number of self-reported comorbidities (angina, arthritis, asthma, cancer, chronic bronchitis, depression, diabetes, epilepsy, heart attack, high blood pressure, intermittent claudication, low blood pressure, Parkinson’s disease, peptic ulcers, pernicious anaemia and stroke
[42]	Community-dwelling	Disability, Nursing Home, Injurious Fall, Mortality	78.4 (5.3)	64.6%	754 (267/487)	-	7.5 years	USA	Transplant center
[9]	Acute coronary syndrome	Mortality	77 (7)	42.69%	342 (196/146)	35% frail, 58% prefrail	Median 8.7 years	Spain	Age, sex, dementia, serum creatinine level, limitation in IADL, NYHA III–IV functional class, Charlson comorbidity index, LVEF ≤ 45%, previous admission due to HF, treatment with beta-blockers, and treatment with ACEI/ARB
[43]	Community-dwelling	Cardiovascular disease	73.6 (6.18)	59.82%	3099 (1245/1854)	-	4.4 years	Italy	Age, diabetes, prior admission for heart failure, prior stroke, peripheral artery disease, admission Killip class 2 or higher, atrial fibrillation, hemoglobin, glomerular filtration rate, left ventricular ejection fraction, and revascularization
[46]	Community-dwelling	Cardiovascular disease	76.2 (5.6)	64%	3818 (1374/2448)	7.9%	8.7 years	Italy, Iceland, USA	Age, sex, presence of chronic obstructive pulmonary disease, alcohol drinking, serum 25-hydroxyvitamin D levels, Geriatric Depression Scale score, waist circumference, presence of diabetes, hypertension, and atrial fibrillation; use of antihypertensive agents, low-dose aspirin, anticoagulant drugs, and statins; systolic blood pressure, diastolic blood pressure, and ankle brachial index; serum high-density lipoproteins, low-density lipoproteins, triglycerides, uric acid, fasting plasma glucose, glycosylated hemoglobin, erythrocyte sedimentation rate, and white blood cells; estimated glomerular filtration rate and smoking status
[51]	Heart failure	Mortality	> 75	49.5%	416 (210/206)	76.0%	1 year	Spain	Age, gender, chronic and acute comorbidities, NYHA, and NT-proBNP
[47]	Community-dwelling	Mortality	Alive 73.15 ± 5.79/Deceased 78.11 ± 7.86	48.11%	921 (478/443)	10.97%	6 years	Taiwan	Age, sex, education, marital status, BMI, smoking, alcohol drinking, physical activity, exercising program, hypertension, diabetes mellitus, heart disease, hyperlipidemia, gout, hyperuricemia, arthritis, osteoporosis, stroke, cataract, fall history, sleep impairment and cognitive function
[48]	Community-dwelling	ADL, Independent ADL, Nursing Home, Falls, Mortality	74.2 (2.8)	100%	786 (0/786)	7.2%	5 years	USA	Age, race, educational level, smoking, and disease-related variables, namely history of congestive heart failure, Mini-Mental State Examination score, Geriatric Depression Scale score, number of adjudicated diseases, ankle-brachial index, and use of diuretics without history of hypertension or congestive heart failure
[49]	Undergoing hemodialysis	Mortality, Hospitalization	67	49%	157 (80/77)	37.6%	3 months	China	Age, dialysis duration, GNRI, serum potassium, URR, and medical history of peripheral vascular disease and chronic pulmonary disease

BMI, Body Mass Index; BP, Blood Pressure; eGFR, estimated Glomerular Filtration Rate; HF, Heart Failure; GOLD, Global Initiative for Chronic Obstructive Lung Disease; COPD, Chronic Obstructive Pulmonary Disease; MMSE, Mini-Mental State Examination; LVEF, Left Ventricular Ejection Fraction; BADL, Basic Activities of Daily Living; IADL, Instrumental Activities of Daily Living; CVD, Cardiovascular Disease; NYHA, New York Heart Association; ACEI/ARB, Angiotensin-Converting Enzyme Inhibitors/Angiotensin Receptor Blockers; GNRI, Geriatric Nutritional Risk Index; URR, Urea Reduction Ratio.

### Weakness

**Fig. 2:**
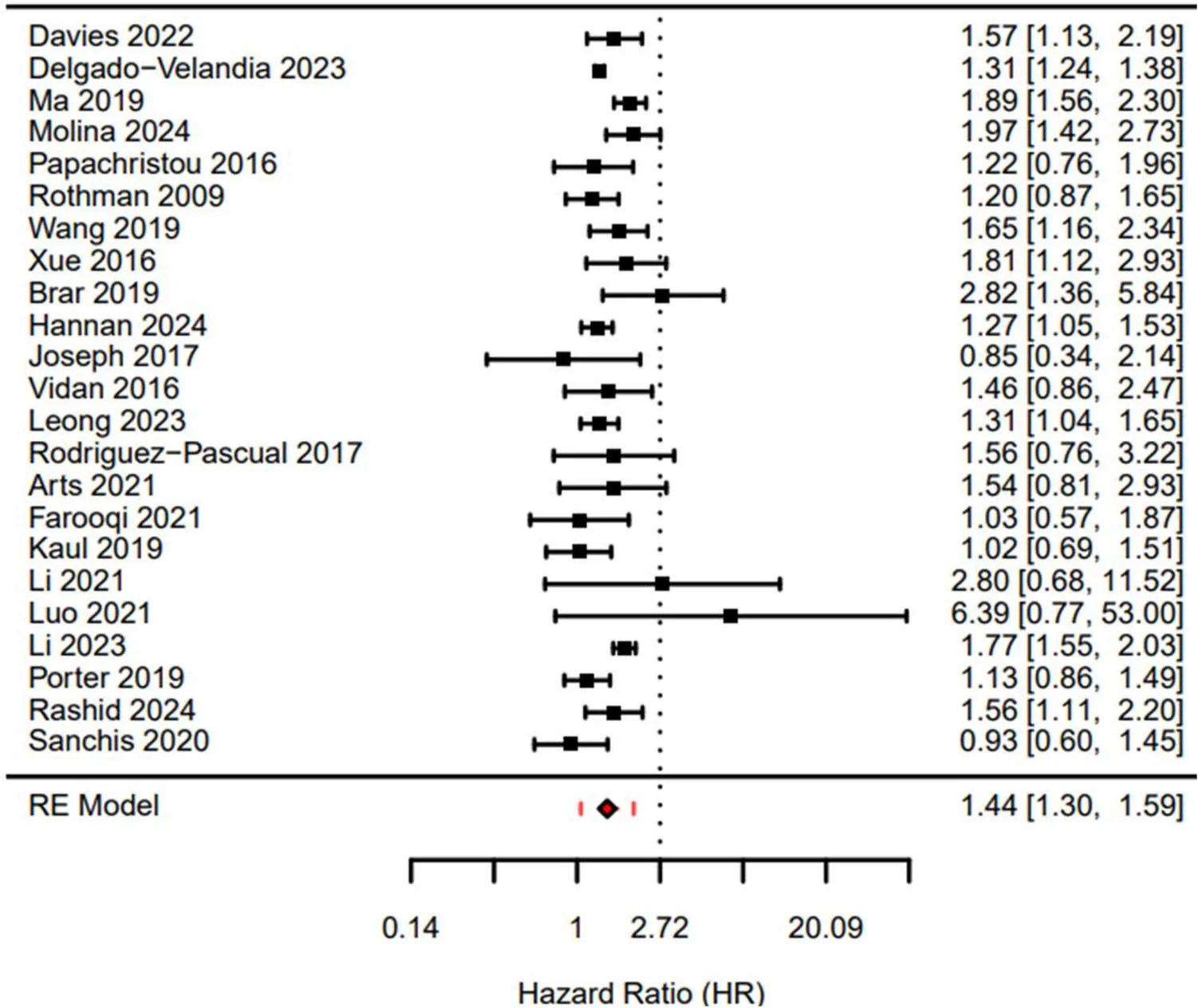
Risk of all-cause mortality due to weakness

Weakness led to a statistical significant effect on all-cause mortality (k = 23; HR: 1.44, 95%CI: 1.30–1.59, p < 0.01, I^2^ = 59%) (Fig. 2). Likewise, a similar effect was demonstrated on cardiovascular mortality (k = 3; HR: 1.45, 95%CI: 1.31–1.60, p < 0.01, I^2^ = 4%) (Figure S1). No statistically significant impact was observed in relation to cardiovascular events (k = 4; HR: 1.12, 95%CI: 0.90–1.40, p = 0.31, I^2^ = 58%) (Figure S2). Likewise, weakness was not associated with hospitalization (k = 3; HR: 1.20, 95%CI: 0.94–1.53, p = 0.15, I^2^ = 35%) (Figure S3) nor falls (k = 3; HR: 0.96, 95%CI: 0.91–1.01, p = 0.15, I^2^ = 0%) (Figure S4). In subgroup analyses, weakness was a contributing factor to all-cause mortality also in community-dwelling individuals (k = 8; HR: 1.55, 95%CI: 1.34–1.78, p < 0.01, I^2^ = 65%) (Figure S5) and those with HF (k = 4; HR: 1.32, 95%CI: 1.09–1.61, p < 0.01, I^2^ = 0%) (Figure S6). Lastly, no changes were shown regarding cardiovascular events in community-dwelling individuals (k = 3; HR: 1.12, 95%CI: 0.89–1.41, p = 0.34, I^2^ = 69%) (Figure S7).

### Slowness

**Fig. 3:**
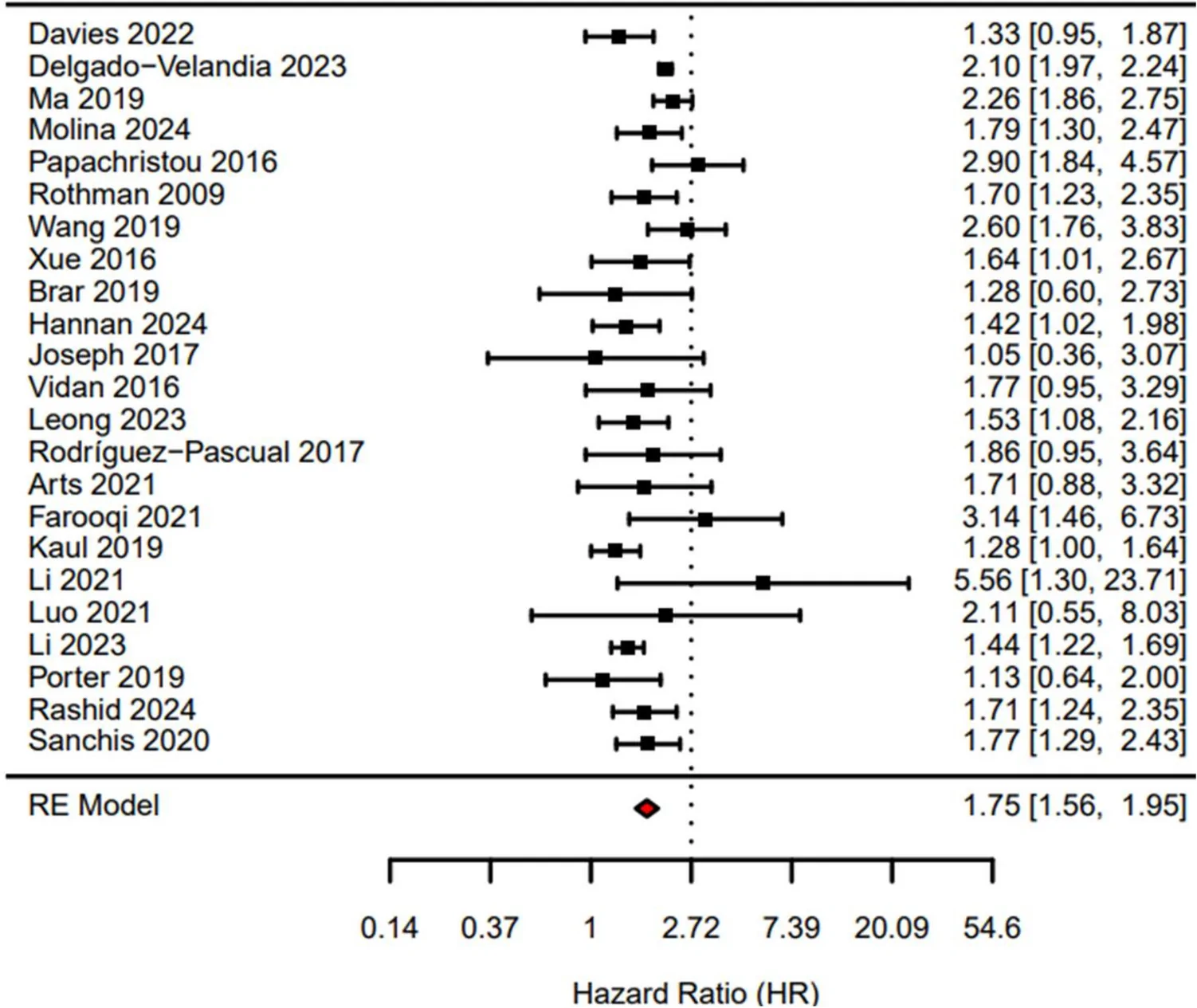
Risk of all-cause mortality due to slowness

Slowness was displayed as significant prognostic contributor to elevated all-cause mortality (k = 23; HR: 1.75, 95%CI: 1.56–1.95, p < 0.01, I^2^ = 59%) (Fig. 3), cardiovascular mortality (k = 3; HR: 1.97, 95%CI: 1.29–3.00, p < 0.01, I2 = 85%) (Figure S8), and hospitalization (k = 3; HR: 1.46, 95%CI: 1.15–1.85, p < 0.01, I2 = 42%) (Figure S9). No statistically significant impact was demonstrated related to cardiovascular events (k = 4; HR: 1.16, 95%CI: 1.00–1.34, p = 0.05, I2 = 0%) (Figure S10) or falls (k = 3; HR: 1.13, 95%CI: 0.81–1.59, p = 0.47, I2 = 0%) (Figure S11). In subgroup analyses, slowness increased all-cause mortality risk ~ two-fold in community-dwelling individuals (k = 8; HR: 1.99, 95%CI: 1.73–2.30, p < 0.01, I2 = 57%) (Figure S12). Similar results were revealed in patients with HF (k = 4; HR: 1.56, 95%CI: 1.28–1.91, p < 0.01, I^2^ = 0%) (Fig. 13). Slowness was not a significant prognostic factor of cardiovascular events in community-dwelling individuals (k = 3; HR: 1.15, 95%CI: 0.99–1.33, p = 0.07, I2 = 0%) (Figure S14).

### Exhaustion

**Fig. 4:**
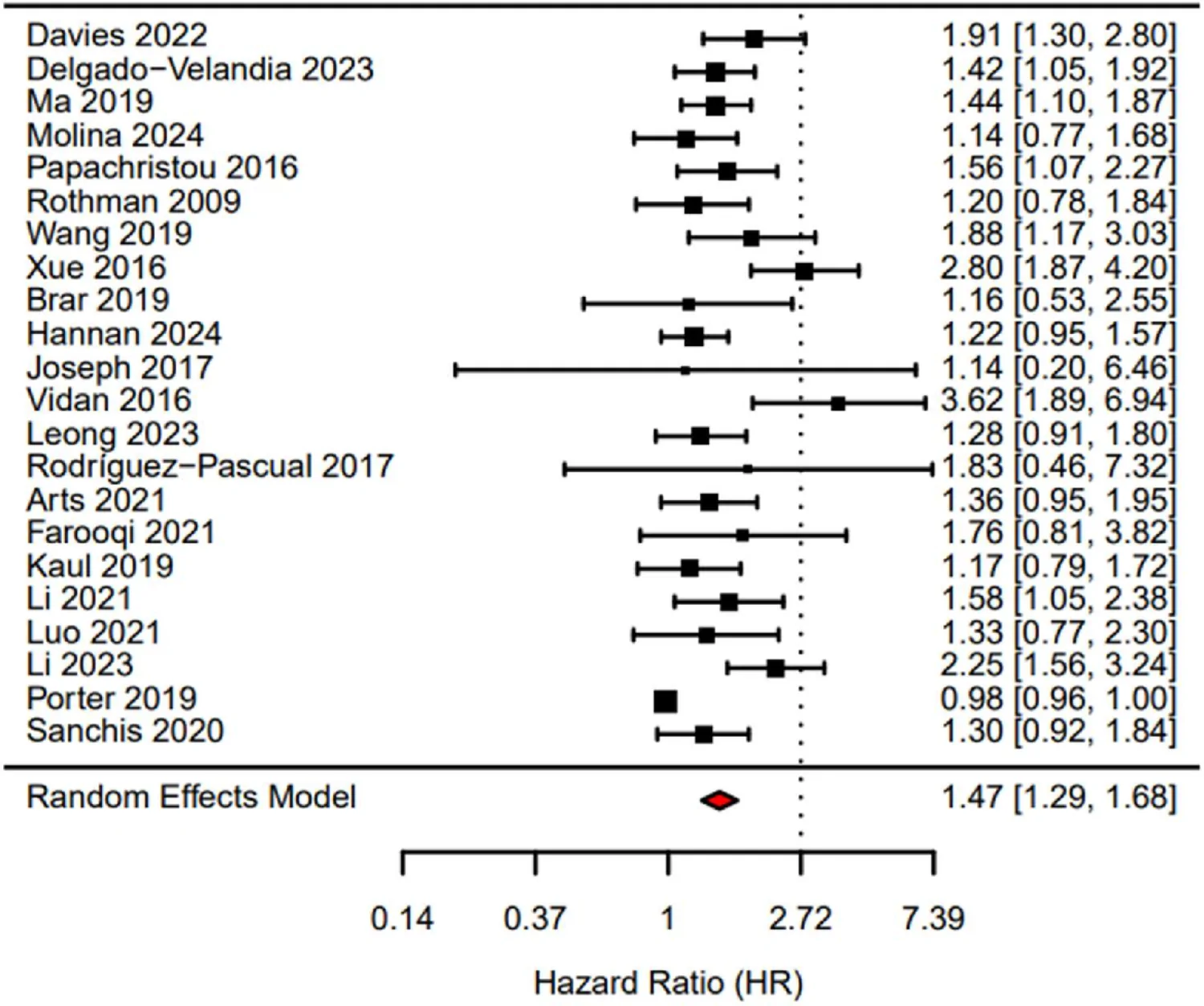
Risk of all-cause mortality due to exhaustion

Elevated risk for all-cause mortality (k = 22; HR: 1.47, 95%CI: 1.29–1.68, p < 0.01, I2 = 71%) (Fig. 4), cardiovascular events (k = 4; HR: 1.33, 95%CI: 1.11–1.58, p < 0.01, I2 = 3%) (Figure S15), and hospitalization (k = 3; HR: 1.25, 95%CI: 1.09–1.44, p < 0.01, I2 = 0%) (Figure S16) was demonstrated due to exhaustion. However, no statistical changes were observed pertaining to cardiovascular mortality (k = 3; HR: 1.29, 95%CI: 0.99–1.70, p = 0.06, I2 = 76%) (Figure S17). In subgroup analyses, exhaustion was linked to significantly elevated risk of all-cause mortality risk in community-dwelling individuals (k = 8; HR: 1.43, 95%CI: 1.35–1.52, p < 0.01, I2 = 0%) (Figure S18) and patients with HF (k = 4; HR: 1.48, 95%CI: 1.11–1.99, p < 0.01, I2 = 25%) (Figure S19). It also demonstrated a prominent impact on cardiovascular events in community-dwelling individuals (k = 3; HR: 1.29, 95%CI: 1.09–1.53, p < 0.01, I2 = 0%) (Figure S20).

### Physical inactivity

**Fig. 5:**
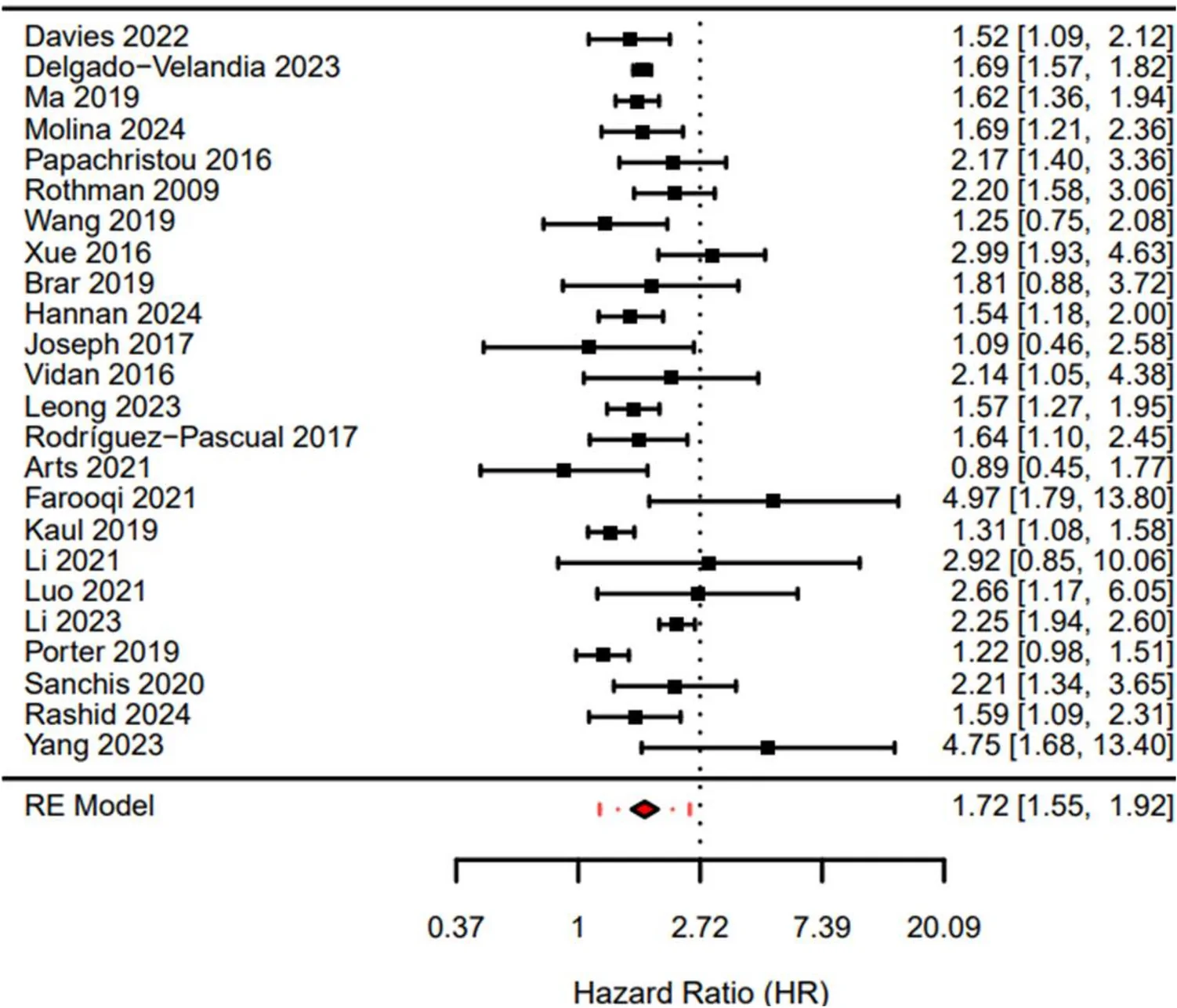
Risk of all-cause mortality due to physical inactivity

Physical inactivity was a prognostic factor for all-cause mortality (k = 24; HR: 1.72, 95%CI: 1.55–1.92, p < 0.01, I^2^ = 64%) (Fig. 5) and cardiovascular mortality (k = 3; HR: 1.26, 95%CI: 1.06–1.48, p < 0.01, I^2^ = 42%) (Figure S21), but not for cardiovascular events (k = 4; HR: 1.10, 95%CI: 0.95–1.26, p = 0.21, I2 = 0%) (Figure S22) or hospitalization (k = 3; HR: 1.20, 95%CI: 0.96–1.51, p = 0.11, I2 = 52%) (Figure S23). In subgroup analyses, physical inactivity was a significant contributor to elevated risk of all-cause mortality risk in community-dwelling individuals (k = 8; HR: 1.71, 95%CI: 1.61–1.82, p < 0.01, I^2^ = 0%) (Figure S24), patients with CKD or hemodialysis (k = 3; HR: 1.98, 95%CI: 1.17–3.36, p = 0.01, I2 = 53%) (Figure S25), and patients with HF (k = 4; HR: 1.59, 95%CI: 1.33–1.90, p < 0.01, I2 = 0%) (Figure S26). No statistical changes were shown relevant to cardiovascular events in community-dwelling individuals (k = 3; HR: 1.09, 95%CI: 0.94–1.26, p = 0.25, I2 = 0%) (Figure S27).

### Unintentional weight loss

**Fig. 6:**
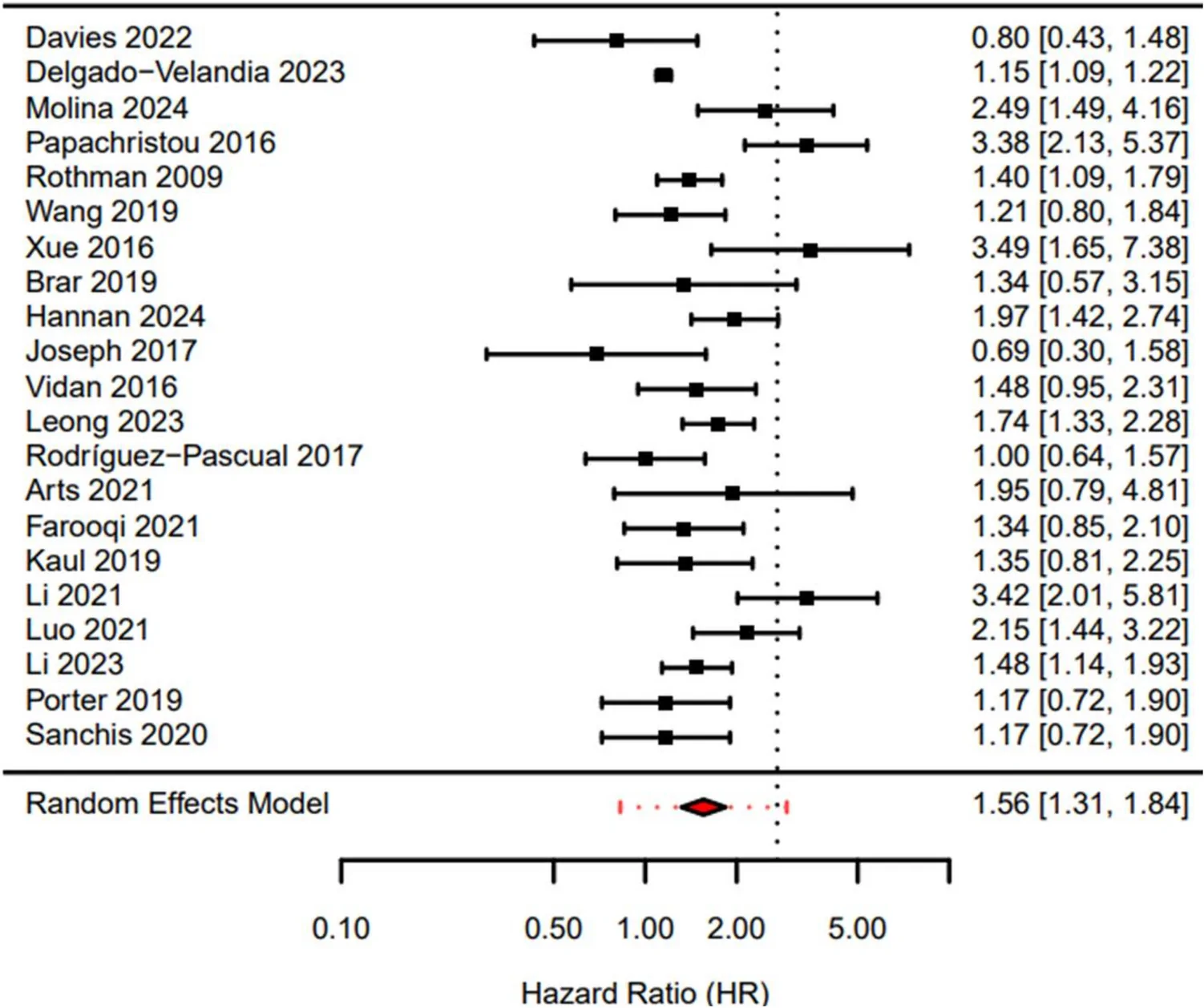
Risk of all-cause mortality due to unintentional weight loss

A prominent prognostic effect was displayed by unintentional weight loss on all-cause mortality (k = 21; HR: 1.56, 95%CI: 1.31–1.84, p < 0.01, I^2^ = 78%) (Fig. 6) and cardiovascular events (k = 4; HR: 1.32, 95%CI: 1.03–1.69, p = 0.03, I^2^ = 0%) (Figure S28). No statistical impact was observed in terms of cardiovascular mortality (k = 3; HR: 1.28, 95%CI: 0.96–1.71, p = 0.09, I2 = 78%) (Figure S29) or hospitalization (k = 3; HR: 1.13, 95%CI: 0.85–1.49, p = 0.41, I2 = 34%) (Figure S30). In subgroup analyses, unintentional weight loss was a prognostic factor of increased risk for all-cause mortality (k = 7; HR: 1.75, 95%CI: 1.13–2.69, p = 0.01, I^2^ = 93%) (Figure S31) and cardiovascular events (k = 3; HR: 1.35, 95%CI: 1.05–1.73, p = 0.02, I2 = 0%) (Figure S32) in community-dwelling individuals. Interestingly, it showed no statistical effect on patients with HF regarding all-cause mortality (k = 4; HR: 1.28, 95%CI: 0.90–1.83, p = 0.16, I^2^ = 59%) (Figure S33).

All results related to main and subgroup analyses are presented in Table S3 and Table S4, respectively.

### Risk of bias results

Based on the risk of bias assessment, most studies scored a high grade, displaying high quality. One study was of moderate quality [29], while another one was of low quality [52]. Details for each domain and overall scores are shown in Tables S5 and S6. The complete GRADE assessment was reported in Supplementary material (Table S7).

### Publication bias and meta-regressions

Publication bias for studies that examined all-cause mortality was demonstrated in terms of “exhaustion” as a frailty criterion (z = 2.14, p = 0.03, b = 0.15). Considering these results, the trim-and-fill method showed an adjusted HR (95%CI) value of 1.47 (1.29–1.68) which is identical to the effect size of the main result. Details for each criterion is shown in Table S8. Furthermore, our meta-regressions revealed that age was a significant moderator of “unintentional weight loss” (z = −2.56, p = 0.01, R^2^ = 78%), while similar findings were shown in relation to body mass index (z = −4.08, p < 0.01, R^2^ = 100%). Finally, taking sex into account through proportion of females in the included studies examining all-cause mortality, it was considered a significant moderator regarding “slowness” (z = 3.08, p < 0.01, R^2^ = 79%) and “exhaustion” (z = 2.22, p = 0.03, R^2^ = 40%) (Table S9). Sensitivity analyses based on studies with higher risk of bias did not alter the results of our main findings (Table S2). We also included funnel plots for each Frailty criteria in Table S2.

## Discussion

As far as we are aware, this the first systematic review and meta-analysis to investigate a potential link between each individual component of the frailty phenotype and mortality. In the 36 studies we extracted, we also analysed the differences observed among studies including adults with different health status. Our results help shed light on the most critical criterion/a pertinent to all-cause or CVD mortality, fall, and hospitalization risk. It should be noted that this study did not directly compare frail versus pre-frail or robust individuals. Rather, our objective was to assess the prognostic significance of the individual components of the Fried phenotype within the context of the broader frailty syndrome. While frailty is defined by the cumulative presence of multiple deficits, understanding the contribution of each component may help clarify which observable impairments most strongly signal vulnerability to adverse outcomes, particularly in settings where full frailty assessment is not feasible.

A Chinese systematic review and meta-analysis of 11 studies and 35,538 older adults showed how frailty phenotype is strictly associated with mortality prediction [53], but they did not study each component of frailty phenotype and their conclusion is based only on the difference between frail and robust older patients in term of mortality. Another Italian systematic review and meta-analysis of 44 studies and 7,026 older adults where they showed that each reduction of 0.1 m/s in gait speed was associated with a 14% increased risk of earlier mortality [54], however this study focused solely on gait speed for predicting mortality, without considering the other components of the frailty phenotype.

A systematic review and meta-analysis of individual frailty criteria provides a complementary perspective to studies focusing on frailty as an integrated syndrome. Rather than replacing this approach, it aims to clarify how single components reflect the underlying construct, identify those most strongly associated with key outcomes, and support earlier risk stratification when full frailty assessment is not feasible, while preserving the cumulative nature of frailty.

In this study weakness was associated with a higher risk of all-cause and cardiovascular mortality. An American study of 19,729 older adults showed how individuals in the weak cohort (based on handgrip results) had a 40% higher risk of mortality relative to persons in the not-weak control cohorts, respectively [55]. Our findings show how weakness was a contributing factor to all-cause mortality in community-dwelling individuals and those with HF. Weakness in the arms and upper body can cause problems with balance and coordination. As handgrip strength weakens, older adults become more susceptible to falling [56]. Added to this, it has been suggested that individuals who are weak often have worse metabolic profiles [57]. For instance, weakness is associated with conditions like myocardial infarction and stroke [58]. Reductions in gait speed and strength have been linked to a higher likelihood of various adverse health outcomes, such as disability, and may also contribute to an elevated risk of falls [59]. Usually an individual with an initial gait speed of 1.5 m/s would experience a 33% reduction in gait speed before reaching 1.0 m/s, a commonly applied threshold for identifying individuals at higher risk of falling [60], at the same time weaker muscle strength were found to be independent risk factors of fall episodes.

In our study slowness and weakness, while usual contributors to frailty, are not significantly associated with falls. Many reasons could explain this result: individuals with slowness or weakness might adopt compensatory strategies, such as using assistive devices or modifying their gait and activity patterns to minimize the risk of falls. These adjustments can reduce their exposure to situations that might lead to a fall. In addition, many people with reduced strength or speed make their environment safer by removing hazards like loose rugs, installing grab bars, or avoiding uneven terrain, which decreases the likelihood of falls despite their physical limitations. Also a previous American study of 763 community-dwelling older people confirmed what we found, in fact they showed how faster gait speed was associated with an increased risk ratio for outdoor falls [61]. This discrepancy between slowness and weakness and falls, may reflect differences in study populations, follow-up duration, outcome definitions, or the limited number of studies reporting falls, potentially reducing statistical power. These findings emphasize the need for cautious interpretation and suggest that future research should further investigate the differential contributions of frailty components to specific adverse outcomes.

Slowness was strongly associated with higher all-cause mortality (Fig. 3), cardiovascular mortality, and increased hospitalization rates. Slowness roughly doubled the risk of all-cause mortality in community-dwelling individuals. Similar findings were observed in patients with HF. Previous literature confirmed our data, a Chinese systematic review and meta-analysis of 9 studies involving 12,901 older adults indicates that the risk of all-cause mortality is 89% higher when comparing the lowest to the highest usual walking speed in adults nearing 65 years of age or older [62];

In this systematic review and meta-analysis increased risk of all-cause mortality (Fig. 4), cardiovascular events, and hospitalization was shown to be associated with exhaustion. It was also associated with a significantly higher risk of all-cause mortality in both community-dwelling individuals and patients with HF. Additionally, it had a notable impact on cardiovascular events in community-dwelling individuals. A Belgian systematic review and meta-analysis of 30 studies and 152,711 older adults showed how exhaustion was found to be prospectively associated with all measured outcomes and frequently studied, leading to a significantly increased risk of mortality, ischemic heart disease, and ADL disability [63]. The European Society of Cardiology also confirmed that individuals experiencing vital exhaustion are more likely to suffer a heart attack [64].

Interestingly, our analysis found that only slowness and exhaustion were significantly associated with hospitalization, whereas weakness and physical inactivity were not. This finding suggests that not all components of the frailty phenotype contribute equally to the risk of hospitalization. Slowness and exhaustion may reflect early impairments in physiological reserve and energy balance, which could predispose individuals to acute decompensation and hospital admission. In contrast, weakness and physical inactivity, while key markers of overall frailty, may exert a more indirect or long-term influence on health outcomes that was not captured within the follow-up periods of the included studies. These results reinforce the importance of examining individual frailty components rather than relying solely on the global phenotype, as each may provide distinct prognostic information and potential targets for intervention.

The mechanism who explained association between exhaustion and mortality are limited, probably this condition has been characterized by diminished hypothalamic–pituitary–adrenal axis responses, low-grade inflammation, and impaired fibrinolytic capacity, all of which have also been independently linked to CVD and higher risk of death [65]. Exhaustion also leads to the depletion of reserve capacity, which is associated with a decline in intrinsic capacity, making the occurrence of negative health outcomes more likely [63].

Physical inactivity was a prognostic factor for both all-cause mortality (Fig. 5) and cardiovascular mortality. Physical inactivity significantly contributed to an increased risk of all-cause mortality also in community-dwelling individuals, patients with CKD or on hemodialysis, and patients with HF. An American study of 538 individuals demonstrated how physical inactivity was linked to a higher risk of both all-cause and cardiac death, while moderate physical activity was associated with a significant reduction in the rates of both overall and cardiac mortality [66].

Unintentional weight loss showed a significant prognostic effect on all-cause mortality (Fig. 6) and cardiovascular events. In subgroup analyses, it was identified as a prognostic factor for an increased risk of both all-cause mortality and cardiovascular events in community-dwelling individuals. An Australian systematic review and meta-analysis with 30 studies, including 1,219,279 older adults, revealed how weight loss was associated with a 59% increase in mortality risk in community-dwelling individuals [67].

Unintentional weight loss increases risk of death and chronic diseases because frequently causes insufficient intake of essential nutrients, leading to malnutrition [68]. Weight loss can result in sarcopenia, which diminishes physical function and raises the risk of falls, fractures, and disability. It can also affect breathing, mobility, and overall quality of life, contributing to worse health outcomes [69]. Additionally, unintentional weight loss is commonly associated with chronic inflammation, which is often seen in conditions such as cancer, heart disease, and infections.

Although some included studies exhibited gender imbalance, we accounted for this through a meta-regression analysis using the proportion of female participants as a moderator. Sex was found to significantly influence the associations between slowness and exhaustion with all-cause mortality (z = 3.08, p < 0.01, R^2^ = 79%; and z = 2.22, p = 0.03, R^2^ = 40%, respectively), suggesting that gender distribution contributed to part of the observed heterogeneity.

Our findings should be interpreted considering the largely overlapping effect sizes observed across frailty components, suggesting that individual criteria reflect a shared underlying frailty construct rather than distinct prognostic entities. Therefore, differences in hazard ratios among components should not be viewed as evidence to prioritize one component over another for clinical screening. Importantly, the available literature did not permit a formal comparison between component-level assessments and the full Fried frailty phenotype, nor an evaluation of incremental predictive performance. While components such as slowness and weakness are consistently associated with mortality across populations and clinical subgroups, they should be considered complementary or pragmatic screening markers rather than substitutes for comprehensive frailty assessment. When feasible, the full Fried phenotype remains the most appropriate tool to capture the multidimensional nature of frailty.

### Strengths and limitations

The strength of this analysis lies in its thorough investigation of individual components of the frailty phenotype at once, offering a better understanding of how each component affects health risks in frail populations. By combining data from multiple studies, it provides valuable insights into the wider impact of frailty on long-term health outcomes, helping to inform more focused interventions for individuals at greater risk. As mentioned before this is the first systematic review and meta-analysis who examined all the frailty criteria and their associations with negative health outcomes.

This systematic review and meta-analysis must be interpreted with some limitations. First, studies were predominantly conducted in higher-income settings in North America, Europe, Australia and East Asia, limiting generalisability to low-income countries. Differences in healthcare systems, socioeconomic conditions, lifestyle exposures and morbidity patterns across populations may further influence the applicability of these findings to underrepresented settings and low- and middle-income countries. In addition, potential selection bias should be considered, as many included cohorts were based on community-dwelling or clinically selected populations, potentially underrepresenting institutionalised individuals, severely frail patients, or participants with limited healthcare access. Furthermore, participants with missing data, cognitive impairment, or incomplete follow-up may have been systematically excluded in some studies, potentially leading to healthier study populations and affecting the magnitude and external validity of the observed associations. Secondly, frailty is a multifaceted condition that may not be fully captured by the frailty criteria alone, this has led to the development of alternative models, such as the Frailty Index, the Clinical Frailty Scale or Multidimensional Prognostic Index. Third, studies among them used different adjustments, which may affect the comparability of results. Most included studies reported association measures only, which precluded formal comparisons of predictive performance. Discrimination and reclassification metrics, such as C-statistics, Net Reclassification Improvement, Integrated Discrimination Improvement and head-to-head modelling comparisons between frailty components were rarely available; therefore, our results reflect differences in associations rather than definitive predictive superiority. Additionally, the comparison of multiple frailty phenotype components across several outcomes introduces potential multiplicity; however, formal corrections for multiple testing were not consistently applied, which may increase the risk of type I error. Lastly, studies included in the meta-analysis had different lengths of follow-up, which may affect the comparability of outcome estimates and could bias the pooled results. In addition, the timing and clinical context of frailty assessment varied across studies, with some evaluating frailty during acute illness or hospitalisation and others in relatively stable community-based populations. This heterogeneity may influence prognostic interpretation, as frailty assessed during acute clinical conditions may partly reflect transient health deterioration rather than baseline vulnerability.Additionally, due to the inclusion of studies with mixed-sex populations that did not report separate analyses, subgroup analyses by sex could not be performed, and meta-regression was used as the most appropriate alternative to explore heterogeneity. Another limitation is the heterogeneity in the operationalization and assessment of frailty components across included studies. Grip strength, gait speed, exhaustion, and weight loss were measured using different instruments, cutoffs, and assessment methods, including both self-reported and objective measures, potentially contributing to variability in the observed associations.

Although the Fried frailty phenotype is validated as a syndromic construct, our approach of analyzing its individual components carries inherent conceptual limitations. Each criterion may stem from distinct physiological or pathological processes not necessarily specific to frailty, which could complicate interpretation. Nevertheless, this study provides novel insights by quantitatively comparing the prognostic significance of all five Fried components within a single analytical framework. While associations between specific measures such as gait speed or grip strength and mortality are well established, previous studies have typically examined these variables in isolation. By synthesizing evidence from 36 studies, our analysis offers a comprehensive evaluation of how each component contributes to adverse outcomes, thereby enhancing understanding of frailty heterogeneity and supporting more focused approaches to early identification and intervention.

## Conclusions

In conclusion, our systematic review and meta-analysis demonstrates that the Fried frailty phenotype represents a clinically meaningful composite syndrome associated with adverse health outcomes, including all-cause mortality, cardiovascular events, hospitalization, and falls. Individual components of this phenotype, particularly weakness, slowness, exhaustion, physical inactivity, and unintentional weight loss, should be considered as pragmatic screening indicators that reflect the presence of the underlying multidimensional frailty construct rather than isolated risk factors. Across 36 studies, these associations were consistently observed in both community-dwelling adults and patients with chronic conditions.

These findings support the clinical relevance of conceptualizing frailty as an integrated biological and functional syndrome, while using its individual components primarily for early identification and risk stratification in routine practice. In particular, simple bedside measures such as gait speed and grip strength may serve as practical screening tools that operationalize the broader frailty phenotype.

Recognizing frailty as a unified construct and detecting its component deficits before full clinical manifestation could enhance preventive care and guide targeted interventions in clinical practice. Future research should prioritize interventional and multicenter studies to determine whether addressing the frailty phenotype as a whole, alongside its measurable components used for screening, can improve clinical outcomes and inform decision-making in geriatric and chronic disease populations.

## Supplementary Information

Below is the link to the electronic supplementary material.

Supplementary file1 (TIFF 3257 KB)

Supplementary file2 (TIFF 3288 KB)

Supplementary file3 (TIFF 2639 KB)

Supplementary file4 (TIFF 3465 KB)

Supplementary file5 (TIFF 2875 KB)

Supplementary file6 (TIFF 2965 KB)

Supplementary file7 (TIFF 4876 KB)

Supplementary file8 (TIFF 2772 KB)

Supplementary file9 (TIFF 3081 KB)

Supplementary file10 (TIFF 2869 KB)

Supplementary file11 (TIFF 2975 KB)

Supplementary file12 (TIFF 3063 KB)

Supplementary file13 (TIFF 3727 KB)

Supplementary file14 (TIFF 3016 KB)

Supplementary file15 (TIFF 2891 KB)

Supplementary file16 (TIFF 4475 KB)

Supplementary file17 (TIFF 2658 KB)

Supplementary file18 (TIFF 3493 KB)

Supplementary file19 (DOCX 5510 KB)

Supplementary file20 (DOCX 14 KB)

Supplementary file21 (DOCX 509 KB)

Supplementary file22 (TIFF 2715 KB)

Supplementary file23 (DOCX 19 KB)

Supplementary file24 (DOCX 14 KB)

Supplementary file25 (DOCX 16 KB)

Supplementary file26 (TIFF 3191 KB)

Supplementary file27 (DOCX 25 KB)

Supplementary file28 (DOCX 20 KB)

Supplementary file29 (DOCX 22 KB)

Supplementary file30 (DOCX 19 KB)

Supplementary file31 (TIFF 2486 KB)

Supplementary file32 (TIFF 4721 KB)

Supplementary file33 (TIFF 4249 KB)

Supplementary file34 (TIFF 3034 KB)

Supplementary file35 (TIFF 2666 KB)

Supplementary file36 (TIFF 3279 KB)

Supplementary file37 (TIFF 2720 KB)

Supplementary file38 (TIFF 3051 KB)

Supplementary file39 (TIFF 2710 KB)

Supplementary file40 (TIFF 2999 KB)

Supplementary file41 (TIFF 2825 KB)

Supplementary file42 (TIFF 3523 KB)

Supplementary file43 (TIFF 4532 KB)

## Data Availability

Data is available upon request.
