# Supplementary material for: The prognostic value of frailty criteria in older adults: a systematic review and meta-analysis
Source: Aging Clin Exp Res. 2026 Aug 3;38(1):223. doi: 10.1007/s40520-026-03458-5 (PMC13736367; doi:10.1007/s40520-026-03458-5)
Supplement: Supplementary file 1 [file 40520_2026_3458_MOESM19_ESM.docx]

**Figure S1.** Risk of cardiovascular mortality due to weakness.

**Figure S2.** Risk of cardiovascular events due to weakness.

**Figure S3.** Risk of hospitalization due to weakness.

**Figure S4.** Risk of falls due to weakness.

**Figure S5.** Risk of all-cause mortality in community-dwelling older adults due to weakness.

**Figure S6.** Risk of all-cause mortality in patients with heart failure due to weakness.

**Figure S7.** Risk of cardiovascular events in community-dwelling older adults due to weakness.

**Figure S8.** Risk of cardiovascular mortality due to slowness.

**Figure S9.** Risk of hospitalization due to slowness.

**Figure S10.** Risk of cardiovascular events due to slowness.

**Figure S11.** Risk of falls due to slowness.

**Figure S12.** Risk of all-cause mortality in community-dwelling older adults due to slowness.

**Figure S13.** Risk of all-cause mortality in patients with heart failure due to slowness.

**Figure S14.** Risk of cardiovascular events in community-dwelling older adults due to slowness.

**Figure S15.** Risk of cardiovascular events due to exhaustion.

**Figure S16.** Risk of hospitalization due to exhaustion.

**Figure S17.** Risk of cardiovascular mortality due to exhaustion.

**Figure S18.** Risk of all-cause mortality in community-dwelling older adults due to exhaustion.

**Figure S19.** Risk of all-cause mortality in patients with heart failure due to exhaustion.

**Figure S20.** Risk of cardiovascular events in community-dwelling older adults due to exhaustion.

**Figure S21.** Risk of cardiovascular mortality due to physical inactivity.

**Figure S22.** Risk of cardiovascular events due to physical inactivity.

**Figure S23.** Risk of hospitalization due to physical inactivity.

**Figure S24.** Risk of all-cause mortality in community-dwelling older adults due to physical inactivity.

**Figure S25.** Risk of all-cause mortality in patients with chronic kidney disease and or undergoing hemodialysis due to physical inactivity.

**Figure S26.** Risk of all-cause mortality in patients with heart failure due to physical inactivity.

**Figure S27.** Risk of cardiovascular events in community-dwelling older adults due to physical inactivity.

**Figure S28.** Risk of cardiovascular events due to unintentional weight loss.

**Figure S29.** Risk of cardiovascular mortality due to unintentional weight loss.

**Figure S30.** Risk of hospitalization due to unintentional weight loss.

**Figure S31.** Risk of all-cause mortality in community-dwelling older adults due to unintentional weight loss.

**Figure S32.** Risk of cardiovascular events in community-dwelling older adults due to unintentional weight loss.

**Figure S33.** Risk of all-cause mortality in patients with heart failure due to unintentional weight loss.

**Table S1.** Search terms employed in the screening of the literature search.

| **Database** | **Search terms** |
| --- | --- |
|  |  |
| PubMed | (Fried AND frail*) AND (hospitali* OR admission* OR mortality OR survival OR prognos* OR death OR fall*) |
| Cochrane Library | (Fried AND frail*) AND (hospitali* OR admission* OR mortality OR survival OR prognos* OR death OR fall*) |
| Web of Science | (Fried AND frail*) AND (hospitali* OR admission* OR mortality OR survival OR prognos* OR death OR fall*) |
| Scopus | (Fried AND frail*) AND (hospitali* OR admission* OR mortality OR survival OR prognos* OR death OR fall*) |

**Table S2.** Meta-regression analyses showing the association strength of age and BMI with the prevalence of geriatric markers by sarcopenia status.

| **Moderators** | **ADL** | | **IADL** | | **MNA** | | **GDS** | | **MMSE** | | **Living alone** | |
| --- | --- | --- | --- | --- | --- | --- | --- | --- | --- | --- | --- | --- |
|  | **Coef (SE)** | **P value** | **Coef (SE)** | **P value** | **Coef (SE)** | **P value** | **Coef (SE)** | **P value** | **Coef (SE)** | **P value** | **Coef. (SE)** | **P value** |
| **Age in those with sarcopenia** | -0.15 (0.21) | 0.51 | 0.31 (0.19) | 0.12 | 0.003 (0.04) | 0.94 | 0.11 (0.04) | 0.009* | 0.02 (0.04) | 0.65 | -0.02 (0.04) | 0.62 |
| **Age in those with no sarcopenia** | -0.06 (0.18) | 0.76 | 0.16 (0.20) | 0.45 | 0.01 (0.03) | 0.69 | 0.08 (0.04) | 0.06 | -0.002 (0.05) | 0.97 | -0.04 (0.04) | 0.35 |
| **BMI in those with sarcopenia** | 0.30 (0.40) | 0.47 | -0.04 (0.33) | 0.92 | 0.22 (0.11) | 0.06 | -0.11 (0.10) | 0.32 | 0.15 (0.18) | 0.40 | -0.02 (0.15) | 0.88 |
| **BMI in those with no sarcopenia** | 0.18 (0.34) | 0.61 | 0.09 (0.37) | 0.81 | 0.26 (0.13) | 0.05 | -0.03 (0.13) | 0.82 | 0.03 (0.20) | 0.89 | -0.02 (0.16) | 0.92 |
| **Proportion of females in those with sarcopenia** | -0.003 (0.064) | 0.96 | -0.010 (0.018) | 0.57 | -0.007 (0.015) | 0.65 | 0.006 (0.016) | 0.73 | 0.016 (0.042) | 0.70 | 0.005 (0.016) | 0.74 |
| **Proportion of females in those with no sarcopenia** | 0.001 (0.063) | 0.98 | 0.019 (0.025) | 0.45 | 0.0004 (0.016) | 0.98 | -0.016 (0.018) | 0.37 | -0.036 (0.039) | 0.35 | -0.001 (0.021) | 0.96 |

*A p value < 0.05 indicates significance

**Table S3.** Publication bias analysis.

|  | **Egger’s test, SE (p-value)** | **Trim and fill** |
| --- | --- | --- |
| **ADL** | -12.10 (5.95) (p=0.07) | 0.104 (0.02-0.522) [6 L] |
| **IADL** | -14.47 (5.84) (p=0.05) | 0.185 (0.056-0.610) [1 L] |
| **MNA** | -0.41 (0.60) (p=0.50) | Unchanged |
| **GDS** | 0.88 (0.69) (p=0.22) | Unchanged |
| **MMSE** | -0.45 (0.63) (p=0.48) | Unchanged |
| **Living alone** | 0.24 (0.13) (p=0.08) | Unchanged |

**Table S4.** Risk of bias assessment of case-control studies.

|  | A. Selection | | | | B. Comparability | C. Outcome | | | Agency for Healthcare Research and Quality (AHRQ) standards |  |
| --- | --- | --- | --- | --- | --- | --- | --- | --- | --- | --- |
| Studies (year) | 1. Is the case definition adequate | 2. Representativeness of the cases | 3. Selection of Controls | 4.Definition of Controls | 1. Comparability of cases and controls on the basis of the design or analysis | 1. Ascertainment of exposure | 2. Same method of ascertainment for cases and controls | 3. Non- Response Rate |  | Score |
| Kameyama 2022 | ★ | ★ |  |  |  | ★ |  |  | Poor Quality | 3 |
| Suzan 2022 | ★ | ★ | ★ | ★ | ★★ | ★ | ★ |  | Good Quality | 8 |
| Yang 2023 | ★ | ★ | ★ | ★ | ★★ | ★ | ★ |  | Good Quality | 8 |

**Table S5.** Risk of bias assessment of cross-sectional studies.

|  | A.B2:E3 Selection | | | | B. Comparability | C. Outcome | |  |  |
| --- | --- | --- | --- | --- | --- | --- | --- | --- | --- |
| Studies (year) | 1. Representativeness of the cases | 2. Sample size | 3. Non-Response rate | 4. Ascertainment of the screening/surveillance tool: | 1.The potential confounders were investigated by subgroup analysis or multivariable analysis. | 1. Assessment of outcome | 2. Statistical test | Agency for Healthcare Research and Quality (AHRQ) standards | Score |
| Asaoka 2024 | ★ | ★ | ★ | ★★ | ★ | ★★ | ★ | Good quality | 9 |
| Ferring 2024 | ★ | ★ | ★ | ★★ | ★ | ★★ | ★ | Good quality | 9 |
| Bellanti 2023 | ★ |  |  | ★ ★ | ★ | ★ ★ | ★ | Fair quality | 7 |
| Bian 2023 | ★ |  |  | ★ ★ | ★ | ★ ★ | ★ | Fair quality | 7 |
| Chen 2021 | ★ |  |  | ★ ★ | ★ | ★ ★ | ★ | Fair quality | 7 |
| Chew 2020 | ★ |  |  | ★ ★ | ★ | ★ ★ | ★ | Fair quality | 7 |
| Chew 2022 | ★ |  |  | ★ ★ | ★ | ★ ★ | ★ | Fair quality | 7 |
| Darroch 2022 | ★ |  |  | ★ ★ | ★ | ★ ★ | ★ | Fair quality | 7 |
| Fan 2022 | ★ | ★ | ★ | ★ ★ | ★ | ★ ★ | ★ | Good quality | 9 |
| Ferrari 2021 | ★ |  |  | ★ ★ | ★ | ★ ★ | ★ | Fair quality | 7 |
| Fonfría‑Vivas 2023 | ★ |  |  | ★ ★ | ★ | ★ ★ | ★ | Fair quality | 7 |
| Formiga 2022 | ★ |  |  | ★ ★ | ★ | ★ ★ | ★ | Fair quality | 7 |
| Fung 2019 | ★ | ★ |  | ★ ★ | ★ | ★ ★ | ★ | Good quality | 8 |
| Hanada 2022 | ★ |  |  | ★ ★ | ★ | ★ ★ | ★ | Fair quality | 7 |
| Hayashi 2019 | ★ | ★ | ★ | ★ ★ | ★ | ★ ★ | ★ | Good quality | 9 |
| He 2022 | ★ |  |  | ★ ★ | ★ | ★ ★ | ★ | Fair quality | 7 |
| Hsu 2021 | ★ |  |  | ★ ★ | ★ | ★ ★ | ★ | Fair quality | 7 |
| Imai 2022 |  |  |  | ★ ★ |  | ★ ★ | ★ | Poor quality | 5 |
| Inoue 2022 | ★ | ★ | ★ | ★ ★ | ★ | ★ ★ | ★ | Good quality | 9 |
| Li 2022 | ★ |  |  | ★ ★ | ★ | ★ ★ | ★ | Fair quality | 7 |
| Jyvakorpi 2020 | ★ |  |  | ★ ★ | ★ | ★ ★ | ★ | Fair quality | 7 |
| Kaluzniak-Szymanowska 2022 | ★ |  |  | ★ ★ | ★ | ★ ★ | ★ | Fair quality | 7 |
| Kimura 2018 | ★ |  |  | ★ ★ | ★ | ★ ★ | ★ | Fair quality | 7 |
| Kirk 2020 | ★ |  |  | ★ ★ | ★ | ★ ★ | ★ | Fair quality | 7 |
| Kugimiya 2022 | ★ |  |  | ★ ★ | ★ | ★ ★ | ★ | Fair quality | 7 |
| Lee 2018 | ★ |  |  | ★ ★ | ★ | ★ ★ | ★ | Fair quality | 7 |
| Li 2022 | ★ |  |  | ★ ★ | ★ | ★ ★ | ★ | Fair quality | 7 |
| Lyu 2022 | ★ |  |  | ★ ★ | ★ | ★ ★ | ★ | Fair quality | 7 |
| Mochizuki 2019 | ★ |  |  | ★ ★ | ★ | ★ ★ | ★ | Fair quality | 7 |
| Moghadam 2023 | ★ |  |  | ★ ★ | ★ | ★ ★ | ★ | Fair quality | 7 |
| Momoki 2017 | ★ |  |  | ★ ★ | ★ | ★ ★ | ★ | Fair quality | 7 |
| Murphy 2023 | ★ |  |  | ★ ★ | ★ | ★ ★ | ★ | Fair quality | 7 |
| Ohta 2023 | ★ |  |  | ★ ★ | ★ | ★ ★ | ★ | Fair quality | 7 |
| Ohtsubo 2022 | ★ | ★ | ★ | ★ ★ | ★ | ★ ★ | ★ | Good quality | 9 |
| Shiba 2023 | ★ |  |  | ★ ★ | ★ | ★ ★ | ★ | Fair quality | 7 |
| Torii 2019 | ★ |  |  | ★ ★ | ★ | ★ ★ | ★ | Fair quality | 7 |
| Tseng 2021 | ★ |  |  | ★ ★ | ★ | ★ ★ | ★ | Fair quality | 7 |
| Umegaki 2020 | ★ |  |  | ★ ★ | ★ | ★ ★ | ★ | Fair quality | 7 |
| Yamada 2021 | ★ |  |  | ★ ★ | ★ | ★ ★ | ★ | Fair quality | 7 |
| Zhang 2022a | ★ |  |  | ★ ★ | ★ | ★ ★ | ★ | Fair quality | 7 |
| Zheng 2023 | ★ |  |  | ★ ★ | ★ | ★ ★ | ★ | Fair quality | 7 |
| Zhu 2023a | ★ |  |  | ★ ★ | ★ | ★ ★ | ★ | Fair quality | 7 |

**Table S6.** Risk of bias assessment of cohort studies.

|  | A. Selection | | | | B. Comparability | C. Outcome | | | Agency for Healthcare Research and Quality (AHRQ) standards |  |
| --- | --- | --- | --- | --- | --- | --- | --- | --- | --- | --- |
| Studies (year) | 1. Representativeness of the exposed cohort | 2. Selection of the non-exposed cohort | 3. Ascertainment of exposure | 4. Demonstration that outcome of interest was not present at start of study | 1. Comparability of cohort on the basis of the design or analysis | 1. Assessment of outcome | 2. Was follow-up long enough for outcomes to occur | 3. Adequacy of follow-up of cohorts |  | Score |
| Shiraishi 2024 | ★ | ★ |  |  | ★ | ★ |  |  | Poor Quality | 4 |
| Assantachai 2021 | ★ |  | ★ | ★ | ★ ★ | ★ |  |  | Fair Quality | 6 |
| Brugliera 2023 | ★ |  | ★ | ★ | ★ ★ |  |  |  | Poor Quality | 5 |
| Chan 2016 | ★ | ★ | ★ | ★ | ★ ★ | ★ | ★ | ★ | Good Quality | 9 |
| Choe 2022 | ★ | ★ | ★ | ★ | ★★ | ★ | ★ | ★ | Good Quality | 9 |
| Das 2020 | ★ | ★ | ★ | ★ | ★ ★ | ★ | ★ | ★ | Good Quality | 9 |
| Hai 2017 | ★ |  | ★ | ★ | ★ ★ |  |  |  | Fair Quality | 5 |
| Han 2020 | ★ | ★ | ★ | ★ | ★★ | ★ | ★ | ★ | Good Quality | 9 |
| Kamijo 2018 | ★ | ★ | ★ | ★ | ★★ | ★ |  |  | Good Quality | 7 |
| Kanaya 2023 | ★ | ★ | ★ | ★ | ★★ | ★ | ★ | ★ | Good Quality | 9 |
| Kasahara 2023 | ★ | ★ | ★ | ★ | ★★ | ★ | ★ | ★ | Good Quality | 9 |
| Lee 2022 | ★ | ★ | ★ | ★ | ★★ | ★ | ★ | ★ | Good Quality | 9 |
| Lee 2022a | ★ | ★ | ★ |  | ★★ | ★ |  | ★ | Fair Quality | 7 |
| Matsumoto 2020 | ★ | ★ | ★ | ★ | ★★ | ★ |  |  | Fair Quality | 7 |
| Pang 2021 | ★ | ★ | ★ | ★ | ★★ | ★ |  |  | Fair Quality | 7 |
| Rossi 2021 | ★ | ★ | ★ | ★ | ★★ | ★ | ★ | ★ | Good Quality | 9 |
| Sazlina 2020 | ★ | ★ | ★ | ★ | ★★ | ★ |  |  | Fair Quality | 7 |
| Shimada 2021 | ★ | ★ | ★ | ★ | ★★ | ★ |  |  | Fair Quality | 7 |
| Shin 2022 | ★ | ★ | ★ | ★ | ★★ | ★ | ★ | ★ | Good Quality | 9 |
| Sobestiansky 2019 | ★ | ★ | ★ | ★ | ★★ | ★ |  |  | Fair Quality | 7 |
| Tay 2015 | ★ | ★ | ★ | ★ | ★★ | ★ |  |  | Fair Quality | 7 |
| Ueoka 2022 | ★ | ★ | ★ | ★ | ★★ | ★ |  |  | Fair Quality | 7 |
| Wang 2023 | ★ | ★ | ★ | ★ | ★★ | ★ |  |  | Fair Quality | 7 |
| Yagi 2021 | ★ | ★ | ★ | ★ | ★★ | ★ | ★ | ★ | Good Quality | 9 |
| Yoshimura 2018 | ★ | ★ | ★ | ★ | ★★ | ★ | ★ |  | Good Quality | 8 |
| Zhang 2019 | ★ | ★ | ★ | ★ | ★★ | ★ |  |  | Fair Quality | 7 |
| Xia 2023 | ★ |  | ★ | ★ | ★★ |  |  |  | Poor Quality | 5 |

**Table S7.** Meta-regression analyses of all-cause mortality based on age, body mass index, and proportion of females.

| **All-cause mortality** | | | | | | | | |
| --- | --- | --- | --- | --- | --- | --- | --- | --- |
| **Age** | | | | | | | | |
| Outcome | β | SE | z | P | 95% confidence interval | tau^2^ | I^2^ | R^2^ |
| Weakness | 0.0152 | 0.0304 | 0.5010 | 0.6164 | -0.0443 – 0.0747 | 0.0320 | 44% | 4% |
| Slowness | -0.0013 | 0.0819 | -0.0154 | 0.9877 | -0.1618 – 0.1593 | 0.6411 | 97% | 0% |
| Exhaustion | 0.0402 | 0.0291 | 1.3818 | 0.1670 | - | 0.0323 | 44% | 24% |
| Physical inactivity | -0.0292 | 0.0540 | -0.5414 | 0.5882 | -0.1351 – 0.0766 | 0.1506 | 65% | 0% |
| Unintentional weight loss | -0.0894 | 0.0349 | -2.5626 | 0.01* | -0.1578 – 0.0210 | 0.0149 | 11% | 78% |
| **Body mass index** | | | | | | | | |
| Outcome | β | SE | z | P | 95% confidence interval | tau^2^ | I^2^ | R^2^ |
| Weakness | 0.0162 | 0.0322 | 0.5018 | 0.6158 | -0.0470 – 0.0793 | 0 | 0% | 4% |
| Slowness | 0.0809 | 0.1723 | 0.4693 | 0.6389 | -0.2569 – 0.4186 | 0.9946 | 97% | 0% |
| Exhaustion | 0.0251 | 0.0292 | 0.8588 | 0.3905 | - | 0 | 0% | 0% |
| Physical inactivity | 0.0058 | 0.0840 | 0.0686 | 0.9453 | -0.1589 – 0.1704 | 0.1138 | 55% | 0% |
| Unintentional weight loss | -0.1400 | 0.0343 | -4.0773 | <0.01* | -0.2074 – -0.0727 | 0 | 0% | 100% |
| **Proportion of females** | | | | | | | | |
| Outcome | β | SE | z | P | 95% confidence interval | tau^2^ | I^2^ | R^2^ |
| Weakness | 0.0012 | 0.0086 | 0.1393 | 0.8892 | -0.0156 – 0.0180 | 0.0358 | 37% | 0% |
| Slowness | 0.0247 | 0.0080 | 3.0806 | <0.01* | 0.0090 – 0.0404 | 0.0354 | 61% | 79% |
| Exhaustion | 0.0126 | 0.0057 | 2.2226 | 0.03* | 0.0015 – 0.0238 | 0.0210 | 34% | 40% |
| Physical inactivity | 0.0021 | 0.0121 | 0.1739 | 0.8620 | - | 0.1398 | 68% | 0% |
| Unintentional weight loss | -0.0028 | 0.0101 | -0.2749 | 0.7834 | - | 0.0656 | 35% | 0% |

**Table S8** characteristic of analyses performed; Funnel plots for each frailty criterion; Sensitivity analyses based on studies with higher risk of bias (exclusion of Joseph et al. 2017 and Leong et al. 2023)

**Characteristic of analyses performed**

Database used to perform the analysis: Excel

Scripts used in the software to perform the statistical analysis: meta package in R

Packages used for each type of analysis and R file in RMD, with the analyses performed:
install.packages("meta")

library(meta)

data <- data.frame(

study = c(),

hr = c(),

ci_lb = c(),

ci_ub = c())

data$logHR <- log(data$hr)

data$seTE <- (log(data$ci_ub) - log(data$ci_lb)) / (2 * 1.96)

meta <- metagen(TE = logHR, seTE = seTE, studlab = study, sm = "HR", data = data)

summary(meta)

dev.new()

forest(meta,

xlab = "Hazard Ratio",

ylim = c(),

at = c(),

digits = 2,

col.diamond = "red",

col.diamond.lines = "black",

main = "RE Model")

**Funnel plots for each frailty criterion.**

Risk of all-cause mortality due to weakness

Risk of all-cause mortality due to slowness

Risk of all-cause mortality due to exhaustion

Risk of all-cause mortality due to physical inactivity

Risk of all-cause mortality due to unintentional weight loss

**Sensitivity analyses based on studies with higher risk of bias (exclusion of Joseph et al. 2017 and Leong et al. 2023)**

Risk of all-cause mortality due to weakness 🡪 HR: 1.43, 95%CI 1.28 – 1.59, I^2^ = 49.6%, p<0.01

Risk of all-cause mortality due to slowness 🡪 HR: 1.79, 95%CI 1.59 – 2.02, I^2^ = 63.8%, p<0.01

Risk of all-cause mortality due to exhaustion 🡪 HR: 1.48, 95%CI 1.29 – 1.71, I^2^ = 83.7%, p<0.01

Risk of all-cause mortality due to physical inactivity 🡪 HR: 1.74, 95%CI 1.53 – 1.98, I^2^ = 65.8%, p<0.01

Risk of all-cause mortality due to unintentional weight loss 🡪 HR: 1.60, 95%CI 1.34 – 1.91, I^2^ = 75.1%, p<0.01

**Table S9** GRADE assessment based on each frailty criterion

| **Domain** | **Judgment** | **Impact on Certainty** |
| --- | --- | --- |
| Risk of Bias   Weakness  Slowness  Exhaustion  Physical inactivity  Unintentional weight loss | No No No No No | 0 0 0 0 0 |
| Inconsistency   Weakness  Slowness  Exhaustion  Physical inactivity  Unintentional weight loss | Serious Serious Non-serious Non-serious Non-serious | -1 (I² = 65%) -1 (I² = 57%) 0 0 -2 (I² = 93%) |
| Indirectness   Weakness  Slowness  Exhaustion  Physical inactivity  Unintentional weight loss | No No No No No | 0 0 0 0 0 |
| Imprecision   Weakness  Slowness  Exhaustion  Physical inactivity  Unintentional weight loss | No No No No Serious | 0 0 0 0 -1 (95%CI of 1.13 – 2.69) |
| Publication Bias   Weakness  Slowness  Exhaustion  Physical inactivity  Unintentional weight loss | No No Serious No No | 0 0 -1 (p=0.03) 0 0 |
| Large Effect Size   Weakness  Slowness  Exhaustion  Physical inactivity  Unintentional weight loss | Large Very large Large Large Large | 0 +1 (mean HR of 1.99) 0 0 0 |
| Dose-Response Gradient   Weakness  Slowness  Exhaustion  Physical inactivity  Unintentional weight loss | N/A | N/A |
| All Plausible Confounding  Weakness  Slowness  Exhaustion  Physical inactivity  Unintentional weight loss | None None None None None | 0 0 0 0 0 |
| Overall Certainty  Weakness  Slowness  Exhaustion  Physical inactivity  Unintentional weight loss | Low (⊕⊕) Low (⊕⊕) Low (⊕⊕) Low (⊕⊕) Low (⊕⊕) | - |
