# Supplementary material for: The prognostic value of frailty criteria in older adults: a systematic review and meta-analysis
Source: Aging Clin Exp Res. 2026 Aug 3;38(1):223. doi: 10.1007/s40520-026-03458-5 (PMC13736367; doi:10.1007/s40520-026-03458-5)
Supplement: Supplementary file 2 [file 40520_2026_3458_MOESM20_ESM.docx]

**Table S1.** Search terms employed in the screening of the literature search.

| **Database** | **Search terms** |
| --- | --- |
|  |  |
| PubMed | (Fried OR frail* OR "gait speed" OR "grip strength" OR weakness OR fatigue OR exhaustion OR "physical activity" OR "weight loss")  AND (hospitali* OR admission* OR mortality OR survival OR prognos* OR death OR fall*) |
| Cochrane Library | (Fried AND frail* OR "gait speed" OR "grip strength" OR weakness OR fatigue OR exhaustion OR "physical activity" OR "weight loss")  AND (hospitali* OR admission* OR mortality OR survival OR prognos* OR death OR fall*) |
| Web of Science | (Fried AND frail* OR "gait speed" OR "grip strength" OR weakness OR fatigue OR exhaustion OR "physical activity" OR "weight loss")  AND (hospitali* OR admission* OR mortality OR survival OR prognos* OR death OR fall*) |
| Scopus | (Fried AND frail* OR "gait speed" OR "grip strength" OR weakness OR fatigue OR exhaustion OR "physical activity" OR "weight loss")  AND (hospitali* OR admission* OR mortality OR survival OR prognos* OR death OR fall*) |
