# Supplementary material for: The prognostic value of frailty criteria in older adults: a systematic review and meta-analysis
Source: Aging Clin Exp Res. 2026 Aug 3;38(1):223. doi: 10.1007/s40520-026-03458-5 (PMC13736367; doi:10.1007/s40520-026-03458-5)
Supplement: Supplementary file 3 [file 40520_2026_3458_MOESM21_ESM.docx]

**Table S2** Details of data elaboration (A), Funnel plots for each frailty criterion (B); Sensitivity analyses based on studies with higher risk of bias (exclusion of Joseph et al. 2017 and Leong et al. 2023) (C)

**(A)**

-Database used to perform the analysis: Excel

-Scripts used in the software to perform the statistical analysis: meta package in R

-Packages used for each type of analysis and R file in RMD, with the analyses performed:
install.packages("meta")

library(meta)

data <- data.frame(

study = c(),

hr = c(),

ci_lb = c(),

ci_ub = c())

data$logHR <- log(data$hr)

data$seTE <- (log(data$ci_ub) - log(data$ci_lb)) / (2 * 1.96)

meta <- metagen(TE = logHR, seTE = seTE, studlab = study, sm = "HR", data = data)

summary(meta)

dev.new()

forest(meta,

xlab = "Hazard Ratio",

ylim = c(),

at = c(),

digits = 2,

col.diamond = "red",

col.diamond.lines = "black",

main = "RE Model")

**(B)**

Risk of all-cause mortality due to weakness

Risk of all-cause mortality due to slowness

Risk of all-cause mortality due to exhaustion

Risk of all-cause mortality due to physical inactivity

Risk of all-cause mortality due to unintentional weight loss

**(C)**

Risk of all-cause mortality due to weakness 🡪 HR: 1.43, 95%CI 1.28 – 1.59, I^2^ = 49.6%, p<0.01

Risk of all-cause mortality due to slowness 🡪 HR: 1.79, 95%CI 1.59 – 2.02, I^2^ = 63.8%, p<0.01

Risk of all-cause mortality due to exhaustion 🡪 HR: 1.48, 95%CI 1.29 – 1.71, I^2^ = 83.7%, p<0.01

Risk of all-cause mortality due to physical inactivity 🡪 HR: 1.74, 95%CI 1.53 – 1.98, I^2^ = 65.8%, p<0.01

Risk of all-cause mortality due to unintentional weight loss 🡪 HR: 1.60, 95%CI 1.34 – 1.91, I^2^ = 75.1%, p<0.01
