# Supplementary material for: The prognostic value of frailty criteria in older adults: a systematic review and meta-analysis
Source: Aging Clin Exp Res. 2026 Aug 3;38(1):223. doi: 10.1007/s40520-026-03458-5 (PMC13736367; doi:10.1007/s40520-026-03458-5)
Supplement: Supplementary file 4 [file 40520_2026_3458_MOESM23_ESM.docx]

**Table S3.** Meta-regression analyses showing the association strength of age and BMI with the prevalence of geriatric markers by sarcopenia status.

| **Moderators** | **ADL** | | **IADL** | | **MNA** | | **GDS** | | **MMSE** | | **Living alone** | |
| --- | --- | --- | --- | --- | --- | --- | --- | --- | --- | --- | --- | --- |
|  | **Coef (SE)** | **P value** | **Coef (SE)** | **P value** | **Coef (SE)** | **P value** | **Coef (SE)** | **P value** | **Coef (SE)** | **P value** | **Coef. (SE)** | **P value** |
| **Age in those with sarcopenia** | -0.15 (0.21) | 0.51 | 0.31 (0.19) | 0.12 | 0.003 (0.04) | 0.94 | 0.11 (0.04) | 0.009* | 0.02 (0.04) | 0.65 | -0.02 (0.04) | 0.62 |
| **Age in those with no sarcopenia** | -0.06 (0.18) | 0.76 | 0.16 (0.20) | 0.45 | 0.01 (0.03) | 0.69 | 0.08 (0.04) | 0.06 | -0.002 (0.05) | 0.97 | -0.04 (0.04) | 0.35 |
| **BMI in those with sarcopenia** | 0.30 (0.40) | 0.47 | -0.04 (0.33) | 0.92 | 0.22 (0.11) | 0.06 | -0.11 (0.10) | 0.32 | 0.15 (0.18) | 0.40 | -0.02 (0.15) | 0.88 |
| **BMI in those with no sarcopenia** | 0.18 (0.34) | 0.61 | 0.09 (0.37) | 0.81 | 0.26 (0.13) | 0.05 | -0.03 (0.13) | 0.82 | 0.03 (0.20) | 0.89 | -0.02 (0.16) | 0.92 |
| **Proportion of females in those with sarcopenia** | -0.003 (0.064) | 0.96 | -0.010 (0.018) | 0.57 | -0.007 (0.015) | 0.65 | 0.006 (0.016) | 0.73 | 0.016 (0.042) | 0.70 | 0.005 (0.016) | 0.74 |
| **Proportion of females in those with no sarcopenia** | 0.001 (0.063) | 0.98 | 0.019 (0.025) | 0.45 | 0.0004 (0.016) | 0.98 | -0.016 (0.018) | 0.37 | -0.036 (0.039) | 0.35 | -0.001 (0.021) | 0.96 |

*A p value < 0.05 indicates significance.
