# Supplementary material for: The prognostic value of frailty criteria in older adults: a systematic review and meta-analysis
Source: Aging Clin Exp Res. 2026 Aug 3;38(1):223. doi: 10.1007/s40520-026-03458-5 (PMC13736367; doi:10.1007/s40520-026-03458-5)
Supplement: Supplementary file 5 [file 40520_2026_3458_MOESM24_ESM.docx]

**Table S4.** Publication bias analysis.

|  | **Egger’s test, SE (p-value)** | **Trim and fill** |
| --- | --- | --- |
| **ADL** | -12.10 (5.95) (p=0.07) | 0.104 (0.02-0.522) [6 L] |
| **IADL** | -14.47 (5.84) (p=0.05) | 0.185 (0.056-0.610) [1 L] |
| **MNA** | -0.41 (0.60) (p=0.50) | Unchanged |
| **GDS** | 0.88 (0.69) (p=0.22) | Unchanged |
| **MMSE** | -0.45 (0.63) (p=0.48) | Unchanged |
| **Living alone** | 0.24 (0.13) (p=0.08) | Unchanged |
