# Supplementary material for: The prognostic value of frailty criteria in older adults: a systematic review and meta-analysis
Source: Aging Clin Exp Res. 2026 Aug 3;38(1):223. doi: 10.1007/s40520-026-03458-5 (PMC13736367; doi:10.1007/s40520-026-03458-5)
Supplement: Supplementary file 6 [file 40520_2026_3458_MOESM25_ESM.docx]

**Table S5.** Risk of bias assessment of case-control studies.

|  | A. Selection | | | | B. Comparability | C. Outcome | | | Agency for Healthcare Research and Quality (AHRQ) standards |  |
| --- | --- | --- | --- | --- | --- | --- | --- | --- | --- | --- |
| Studies (year) | 1. Is the case definition adequate | 2. Representativeness of the cases | 3. Selection of Controls | 4.Definition of Controls | 1. Comparability of cases and controls on the basis of the design or analysis | 1. Ascertainment of exposure | 2. Same method of ascertainment for cases and controls | 3. Non- Response Rate |  | Score |
| Kameyama 2022 | ★ | ★ |  |  |  | ★ |  |  | Poor Quality | 3 |
| Suzan 2022 | ★ | ★ | ★ | ★ | ★★ | ★ | ★ |  | Good Quality | 8 |
| Yang 2023 | ★ | ★ | ★ | ★ | ★★ | ★ | ★ |  | Good Quality | 8 |
