# Supplementary material for: The prognostic value of frailty criteria in older adults: a systematic review and meta-analysis
Source: Aging Clin Exp Res. 2026 Aug 3;38(1):223. doi: 10.1007/s40520-026-03458-5 (PMC13736367; doi:10.1007/s40520-026-03458-5)
Supplement: Supplementary file 7 [file 40520_2026_3458_MOESM27_ESM.docx]

**Table S6.** Risk of bias assessment of cross-sectional studies.

|  | A.B2:E3 Selection | | | | B. Comparability | C. Outcome | |  |  |
| --- | --- | --- | --- | --- | --- | --- | --- | --- | --- |
| Studies (year) | 1. Representativeness of the cases | 2. Sample size | 3. Non-Response rate | 4. Ascertainment of the screening/surveillance tool: | 1.The potential confounders were investigated by subgroup analysis or multivariable analysis. | 1. Assessment of outcome | 2. Statistical test | Agency for Healthcare Research and Quality (AHRQ) standards | Score |
| Asaoka 2024 | ★ | ★ | ★ | ★★ | ★ | ★★ | ★ | Good quality | 9 |
| Ferring 2024 | ★ | ★ | ★ | ★★ | ★ | ★★ | ★ | Good quality | 9 |
| Bellanti 2023 | ★ |  |  | ★ ★ | ★ | ★ ★ | ★ | Fair quality | 7 |
| Bian 2023 | ★ |  |  | ★ ★ | ★ | ★ ★ | ★ | Fair quality | 7 |
| Chen 2021 | ★ |  |  | ★ ★ | ★ | ★ ★ | ★ | Fair quality | 7 |
| Chew 2020 | ★ |  |  | ★ ★ | ★ | ★ ★ | ★ | Fair quality | 7 |
| Chew 2022 | ★ |  |  | ★ ★ | ★ | ★ ★ | ★ | Fair quality | 7 |
| Darroch 2022 | ★ |  |  | ★ ★ | ★ | ★ ★ | ★ | Fair quality | 7 |
| Fan 2022 | ★ | ★ | ★ | ★ ★ | ★ | ★ ★ | ★ | Good quality | 9 |
| Ferrari 2021 | ★ |  |  | ★ ★ | ★ | ★ ★ | ★ | Fair quality | 7 |
| Fonfría‑Vivas 2023 | ★ |  |  | ★ ★ | ★ | ★ ★ | ★ | Fair quality | 7 |
| Formiga 2022 | ★ |  |  | ★ ★ | ★ | ★ ★ | ★ | Fair quality | 7 |
| Fung 2019 | ★ | ★ |  | ★ ★ | ★ | ★ ★ | ★ | Good quality | 8 |
| Hanada 2022 | ★ |  |  | ★ ★ | ★ | ★ ★ | ★ | Fair quality | 7 |
| Hayashi 2019 | ★ | ★ | ★ | ★ ★ | ★ | ★ ★ | ★ | Good quality | 9 |
| He 2022 | ★ |  |  | ★ ★ | ★ | ★ ★ | ★ | Fair quality | 7 |
| Hsu 2021 | ★ |  |  | ★ ★ | ★ | ★ ★ | ★ | Fair quality | 7 |
| Imai 2022 |  |  |  | ★ ★ |  | ★ ★ | ★ | Poor quality | 5 |
| Inoue 2022 | ★ | ★ | ★ | ★ ★ | ★ | ★ ★ | ★ | Good quality | 9 |
| Li 2022 | ★ |  |  | ★ ★ | ★ | ★ ★ | ★ | Fair quality | 7 |
| Jyvakorpi 2020 | ★ |  |  | ★ ★ | ★ | ★ ★ | ★ | Fair quality | 7 |
| Kaluzniak-Szymanowska 2022 | ★ |  |  | ★ ★ | ★ | ★ ★ | ★ | Fair quality | 7 |
| Kimura 2018 | ★ |  |  | ★ ★ | ★ | ★ ★ | ★ | Fair quality | 7 |
| Kirk 2020 | ★ |  |  | ★ ★ | ★ | ★ ★ | ★ | Fair quality | 7 |
| Kugimiya 2022 | ★ |  |  | ★ ★ | ★ | ★ ★ | ★ | Fair quality | 7 |
| Lee 2018 | ★ |  |  | ★ ★ | ★ | ★ ★ | ★ | Fair quality | 7 |
| Li 2022 | ★ |  |  | ★ ★ | ★ | ★ ★ | ★ | Fair quality | 7 |
| Lyu 2022 | ★ |  |  | ★ ★ | ★ | ★ ★ | ★ | Fair quality | 7 |
| Mochizuki 2019 | ★ |  |  | ★ ★ | ★ | ★ ★ | ★ | Fair quality | 7 |
| Moghadam 2023 | ★ |  |  | ★ ★ | ★ | ★ ★ | ★ | Fair quality | 7 |
| Momoki 2017 | ★ |  |  | ★ ★ | ★ | ★ ★ | ★ | Fair quality | 7 |
| Murphy 2023 | ★ |  |  | ★ ★ | ★ | ★ ★ | ★ | Fair quality | 7 |
| Ohta 2023 | ★ |  |  | ★ ★ | ★ | ★ ★ | ★ | Fair quality | 7 |
| Ohtsubo 2022 | ★ | ★ | ★ | ★ ★ | ★ | ★ ★ | ★ | Good quality | 9 |
| Shiba 2023 | ★ |  |  | ★ ★ | ★ | ★ ★ | ★ | Fair quality | 7 |
| Torii 2019 | ★ |  |  | ★ ★ | ★ | ★ ★ | ★ | Fair quality | 7 |
| Tseng 2021 | ★ |  |  | ★ ★ | ★ | ★ ★ | ★ | Fair quality | 7 |
| Umegaki 2020 | ★ |  |  | ★ ★ | ★ | ★ ★ | ★ | Fair quality | 7 |
| Yamada 2021 | ★ |  |  | ★ ★ | ★ | ★ ★ | ★ | Fair quality | 7 |
| Zhang 2022a | ★ |  |  | ★ ★ | ★ | ★ ★ | ★ | Fair quality | 7 |
| Zheng 2023 | ★ |  |  | ★ ★ | ★ | ★ ★ | ★ | Fair quality | 7 |
| Zhu 2023a | ★ |  |  | ★ ★ | ★ | ★ ★ | ★ | Fair quality | 7 |
