# Supplementary material for: The prognostic value of frailty criteria in older adults: a systematic review and meta-analysis
Source: Aging Clin Exp Res. 2026 Aug 3;38(1):223. doi: 10.1007/s40520-026-03458-5 (PMC13736367; doi:10.1007/s40520-026-03458-5)
Supplement: Supplementary file 8 [file 40520_2026_3458_MOESM28_ESM.docx]

**Table S7– GRADE assessment based on each frailty criterion.**

| **Domain** | **Judgment** | **Impact on Certainty** |
| --- | --- | --- |
| Risk of Bias   Weakness  Slowness  Exhaustion  Physical inactivity  Unintentional weight loss | No No No No No | 0 0 0 0 0 |
| Inconsistency   Weakness  Slowness  Exhaustion  Physical inactivity  Unintentional weight loss | Serious Serious Non-serious Non-serious Non-serious | -1 (I² = 65%) -1 (I² = 57%) 0 0 -2 (I² = 93%) |
| Indirectness   Weakness  Slowness  Exhaustion  Physical inactivity  Unintentional weight loss | No No No No No | 0 0 0 0 0 |
| Imprecision   Weakness  Slowness  Exhaustion  Physical inactivity  Unintentional weight loss | No No No No Serious | 0 0 0 0 -1 (95%CI of 1.13 – 2.69) |
| Publication Bias   Weakness  Slowness  Exhaustion  Physical inactivity  Unintentional weight loss | No No Serious No No | 0 0 -1 (p=0.03) 0 0 |
| Large Effect Size   Weakness  Slowness  Exhaustion  Physical inactivity  Unintentional weight loss | Large Very large Large Large Large | 0 +1 (mean HR of 1.99) 0 0 0 |
| Dose-Response Gradient   Weakness  Slowness  Exhaustion  Physical inactivity  Unintentional weight loss | N/A | N/A |
| All Plausible Confounding  Weakness  Slowness  Exhaustion  Physical inactivity  Unintentional weight loss | None None None None None | 0 0 0 0 0 |
| Overall Certainty  Weakness  Slowness  Exhaustion  Physical inactivity  Unintentional weight loss | Low (⊕⊕) Low (⊕⊕) Low (⊕⊕) Low (⊕⊕) Low (⊕⊕) | - |
