# Supplementary material for: The prognostic value of frailty criteria in older adults: a systematic review and meta-analysis
Source: Aging Clin Exp Res. 2026 Aug 3;38(1):223. doi: 10.1007/s40520-026-03458-5 (PMC13736367; doi:10.1007/s40520-026-03458-5)
Supplement: Supplementary file 9 [file 40520_2026_3458_MOESM29_ESM.docx]

**Table S8.** Risk of bias assessment of cohort studies.

|  | A. Selection | | | | B. Comparability | C. Outcome | | | Agency for Healthcare Research and Quality (AHRQ) standards |  |
| --- | --- | --- | --- | --- | --- | --- | --- | --- | --- | --- |
| Studies (year) | 1. Representativeness of the exposed cohort | 2. Selection of the non-exposed cohort | 3. Ascertainment of exposure | 4. Demonstration that outcome of interest was not present at start of study | 1. Comparability of cohort on the basis of the design or analysis | 1. Assessment of outcome | 2. Was follow-up long enough for outcomes to occur | 3. Adequacy of follow-up of cohorts |  | Score |
| Shiraishi 2024 | ★ | ★ |  |  | ★ | ★ |  |  | Poor Quality | 4 |
| Assantachai 2021 | ★ |  | ★ | ★ | ★ ★ | ★ |  |  | Fair Quality | 6 |
| Brugliera 2023 | ★ |  | ★ | ★ | ★ ★ |  |  |  | Poor Quality | 5 |
| Chan 2016 | ★ | ★ | ★ | ★ | ★ ★ | ★ | ★ | ★ | Good Quality | 9 |
| Choe 2022 | ★ | ★ | ★ | ★ | ★★ | ★ | ★ | ★ | Good Quality | 9 |
| Das 2020 | ★ | ★ | ★ | ★ | ★ ★ | ★ | ★ | ★ | Good Quality | 9 |
| Hai 2017 | ★ |  | ★ | ★ | ★ ★ |  |  |  | Fair Quality | 5 |
| Han 2020 | ★ | ★ | ★ | ★ | ★★ | ★ | ★ | ★ | Good Quality | 9 |
| Kamijo 2018 | ★ | ★ | ★ | ★ | ★★ | ★ |  |  | Good Quality | 7 |
| Kanaya 2023 | ★ | ★ | ★ | ★ | ★★ | ★ | ★ | ★ | Good Quality | 9 |
| Kasahara 2023 | ★ | ★ | ★ | ★ | ★★ | ★ | ★ | ★ | Good Quality | 9 |
| Lee 2022 | ★ | ★ | ★ | ★ | ★★ | ★ | ★ | ★ | Good Quality | 9 |
| Lee 2022a | ★ | ★ | ★ |  | ★★ | ★ |  | ★ | Fair Quality | 7 |
| Matsumoto 2020 | ★ | ★ | ★ | ★ | ★★ | ★ |  |  | Fair Quality | 7 |
| Pang 2021 | ★ | ★ | ★ | ★ | ★★ | ★ |  |  | Fair Quality | 7 |
| Rossi 2021 | ★ | ★ | ★ | ★ | ★★ | ★ | ★ | ★ | Good Quality | 9 |
| Sazlina 2020 | ★ | ★ | ★ | ★ | ★★ | ★ |  |  | Fair Quality | 7 |
| Shimada 2021 | ★ | ★ | ★ | ★ | ★★ | ★ |  |  | Fair Quality | 7 |
| Shin 2022 | ★ | ★ | ★ | ★ | ★★ | ★ | ★ | ★ | Good Quality | 9 |
| Sobestiansky 2019 | ★ | ★ | ★ | ★ | ★★ | ★ |  |  | Fair Quality | 7 |
| Tay 2015 | ★ | ★ | ★ | ★ | ★★ | ★ |  |  | Fair Quality | 7 |
| Ueoka 2022 | ★ | ★ | ★ | ★ | ★★ | ★ |  |  | Fair Quality | 7 |
| Wang 2023 | ★ | ★ | ★ | ★ | ★★ | ★ |  |  | Fair Quality | 7 |
| Yagi 2021 | ★ | ★ | ★ | ★ | ★★ | ★ | ★ | ★ | Good Quality | 9 |
| Yoshimura 2018 | ★ | ★ | ★ | ★ | ★★ | ★ | ★ |  | Good Quality | 8 |
| Zhang 2019 | ★ | ★ | ★ | ★ | ★★ | ★ |  |  | Fair Quality | 7 |
| Xia 2023 | ★ |  | ★ | ★ | ★★ |  |  |  | Poor Quality | 5 |
