# Supplementary material for: The prognostic value of frailty criteria in older adults: a systematic review and meta-analysis
Source: Aging Clin Exp Res. 2026 Aug 3;38(1):223. doi: 10.1007/s40520-026-03458-5 (PMC13736367; doi:10.1007/s40520-026-03458-5)
Supplement: Supplementary file 10 [file 40520_2026_3458_MOESM30_ESM.docx]

**Table S9.** Meta-regression analyses of all-cause mortality based on age, body mass index, and proportion of females.

| **All-cause mortality** | | | | | | | | |
| --- | --- | --- | --- | --- | --- | --- | --- | --- |
| **Age** | | | | | | | | |
| Outcome | β | SE | z | P | 95% confidence interval | tau^2^ | I^2^ | R^2^ |
| Weakness | 0.0152 | 0.0304 | 0.5010 | 0.6164 | -0.0443 – 0.0747 | 0.0320 | 44% | 4% |
| Slowness | -0.0013 | 0.0819 | -0.0154 | 0.9877 | -0.1618 – 0.1593 | 0.6411 | 97% | 0% |
| Exhaustion | 0.0402 | 0.0291 | 1.3818 | 0.1670 | - | 0.0323 | 44% | 24% |
| Physical inactivity | -0.0292 | 0.0540 | -0.5414 | 0.5882 | -0.1351 – 0.0766 | 0.1506 | 65% | 0% |
| Unintentional weight loss | -0.0894 | 0.0349 | -2.5626 | 0.01* | -0.1578 – 0.0210 | 0.0149 | 11% | 78% |
| **Body mass index** | | | | | | | | |
| Outcome | β | SE | z | P | 95% confidence interval | tau^2^ | I^2^ | R^2^ |
| Weakness | 0.0162 | 0.0322 | 0.5018 | 0.6158 | -0.0470 – 0.0793 | 0 | 0% | 4% |
| Slowness | 0.0809 | 0.1723 | 0.4693 | 0.6389 | -0.2569 – 0.4186 | 0.9946 | 97% | 0% |
| Exhaustion | 0.0251 | 0.0292 | 0.8588 | 0.3905 | - | 0 | 0% | 0% |
| Physical inactivity | 0.0058 | 0.0840 | 0.0686 | 0.9453 | -0.1589 – 0.1704 | 0.1138 | 55% | 0% |
| Unintentional weight loss | -0.1400 | 0.0343 | -4.0773 | <0.01* | -0.2074 – -0.0727 | 0 | 0% | 100% |
| **Proportion of females** | | | | | | | | |
| Outcome | β | SE | z | P | 95% confidence interval | tau^2^ | I^2^ | R^2^ |
| Weakness | 0.0012 | 0.0086 | 0.1393 | 0.8892 | -0.0156 – 0.0180 | 0.0358 | 37% | 0% |
| Slowness | 0.0247 | 0.0080 | 3.0806 | <0.01* | 0.0090 – 0.0404 | 0.0354 | 61% | 79% |
| Exhaustion | 0.0126 | 0.0057 | 2.2226 | 0.03* | 0.0015 – 0.0238 | 0.0210 | 34% | 40% |
| Physical inactivity | 0.0021 | 0.0121 | 0.1739 | 0.8620 | - | 0.1398 | 68% | 0% |
| Unintentional weight loss | -0.0028 | 0.0101 | -0.2749 | 0.7834 | - | 0.0656 | 35% | 0% |
